# Significance of the Overexpression of Substance P and Its Receptor NK-1R in Head and Neck Carcinogenesis: A Systematic Review and Meta-Analysis

**DOI:** 10.3390/cancers13061349

**Published:** 2021-03-17

**Authors:** Miguel Ángel González-Moles, Pablo Ramos-García, Francisco Esteban

**Affiliations:** 1 School of Dentistry, University of Granada, 18010 Granada, Spain; 2 Instituto de Investigación Biosanitaria ibs.GRANADA, 18012 Granada, Spain; 3 WHO Collaborating Group for Oral Cancer, 1211 Geneva, Switzerland; 4 Department of Otorhinolaryngology, Virgen del Rocio University Hospital, 41013 Sevilla, Spain; festebano@us.es

**Keywords:** Substance P, NK-1R, neurokinin, tachykinin, head and neck, neoplasm, squamous cell carcinoma, systematic review, meta-analysis

## Abstract

**Simple Summary:**

Head and neck cancer is the sixth most frequent type of cancer, with more than 600,000 new cases/year, and it is responsible for around 300,000 deaths/year. Substance P (SP) is a peptide of the tachykinin family whose functions are related to a large number of physiological mechanisms in humans. The implications of SP in human carcinogenesis have recently been reported through the stimulation of its receptor NK-1R, or directly through the effects derived from the constitutive activation of NK-1R. With this background, we have shown, through a systematic review and meta-analysis, evidence that the upregulation of SP and NK-1R are oncogenic events involved in head and neck carcinogenesis, probably acting in the early stages of malignization. Our findings also highlight translational opportunities for SP/NK-1R as potential therapeutic targets in head and neck cancer.

**Abstract:**

The objective of our study has been, through a systematic review and meta-analysis, to increase the scientific evidence on the implications of SP and its receptor NK-1R in head and neck carcinogenesis. We searched studies published before May-2020 without date and publication language restrictions (PubMed, Embase, Web of Science, Scopus). We evaluated the quality of the studies included (QUIPS tool). We performed heterogeneity, sensitivity, small-study effects, and subgroup analyses. A total 16 studies and 1308 cases met inclusion criteria. Qualitative evaluation demonstrated that not all studies were performed with the same scientific rigor, finding the greatest risk of bias in the study confounding and prognostic factors measurement domains. Quantitative evaluation showed a greater SP/NK-1R overexpression in malignant head and neck lesions compared to benign lesions (*p* = 0.02), and that expression was observed in malignant salivary gland pathology. Likewise, we found a higher overexpression of NK-1R compared to SP (*p* = 0.02). In conclusion, the results of this systematic review and meta-analysis show evidence that the upregulation of SP and NK-1R are oncogenic events involved in head and neck carcinogenesis, probably acting in the early stages of malignization. In addition, there is evidence of a greater relevance of the upregulation of the NK-1R receptor compared to SP, which highlights the interest in deepening the development of targeted therapies on the receptor. Future studies assessing the relationships between SP/NK-1R among subjects with head and neck tumors could consider the recommendations given in this systematic review and meta-analysis to improve and standardize future research.

## 1. Introduction

Head and neck cancer is the sixth most frequent type of cancer, with more than 600,000 new cases/year, and it is responsible for around 300,000 deaths/year [1,2]. Head and neck squamous cell carcinoma (HNSCC) represents >90% of these malignant neoplasms and constitutes a heterogeneous group of tumors with anatomical, clinical, histopathological, and molecular differences [1,3]. Prediction of the prognosis for individual patients is highly important, given the 5-year survival rate of around 60% [4,5]. The tumor node metastasis—TNM—system is an important prognostic tool in HNSCC, and N+ status is associated with the worst prognosis [6]. Currently, research is focused on the prognostic and therapeutic value of emerging molecular biomarkers that may serve as a complement in clinical practice [7,8]. Accumulated evidence suggests that Substance P (SP)/NK-1R alterations play a key role in head and neck oncogenesis, particularly in laryngeal carcinomas and oral squamous cell carcinomas [9,10,11,12].

SP is a peptide of the tachykinin family whose functions are related to a large number of physiological mechanisms in humans [13]. SP shows a widespread distribution in both the central and peripheral nervous systems, but it is also present in human cells of different lineage (immune cells, liver, lung, placenta, etc.). After binding to the NK-1R receptor, also widely expressed in the body, the SP/NK-1R complex regulates many biological roles—physiological and pathological—implicated in neuronal survival and degeneration, in the regulation of the cardiovascular and arterial systems, in the regulation of respiratory mechanisms, in musculoskeletal and gastric motility, in sensory perception, in salivation, micturition, depression, pain, inflammation, and in cancer [14]. The implications of SP in human carcinogenesis have recently been reported through the stimulation of its receptor NK-1R, or directly through the effects derived from the constitutive activation of NK-1R, which induces proliferative actions, mediated primarily through the MAPK and PI3K oncogenic pathways. Therefore, the universal mitogenic action exerted by SP on tumor cells ay promotes the most representative canonical hallmark of cancer, i.e., sustaining proliferative signaling [15,16,17,18,19]. On the other hand, both SP and NK-1R seem to exert important emergent roles in cancer, e.g., increased cell migration, invasiveness and metastasis, neoangiogenesis and chronic inflammation, cell death evasion and reprogramming energy metabolism [16,20,21,22,23,24,25]. The oncogenic actions of SP/NK-1R have been reported in several human neoplasms, such as acute myeloid leukemia [26], brain tumors [16], breast [23], endometrial [22], or pancreatic [21] cancers. Although the pioneering work by Henning et al. [27] on SP/NK-1R did not provide information on squamous cell carcinomas, research results are available on the importance of alterations of this protein and its receptor in squamous cells carcinomas; in this sense, our research group have reported results in pre-malignant and malignant epithelia of the larynx and oral cavity [10,12,28]. The importance of up-regulation of NK-1R in squamous carcinomas also concerns the possibility of performing a targeted tumor therapy on NK-1R, and there are currently at least 3 phase 4 clinical trials on this topic (NCT00588835; NCT02532634; NCT04134208. Source: clinicaltrials.gov). Among NK-1R antitumor drugs, the inhibitor aprepitant should be highlighted, having shown traslational potential in a broad spectrum of cancers, such as breast, colon, gastric, laryngeal, lung and pancreatic cancers, esophageal squamous cell carcinoma, leukemias, retinoblastoma, neuroblastoma, glioma, osteosarcoma or melanoma [25].

Thus, with this background, the objective of our study has been, through a systematic review and meta-analysis, to increase the scientific evidence on the implications of SP and its receptor NK-1R in head and neck carcinogenesis, evaluating the possibilities of its use as therapeutic targets in the treatment of these tumors through their differential expression.

## 2. Materials and Methods

This systematic review and meta-analysis complied with MOOSE and PRISMA guidelines [29,30] and closely followed the criteria of *Cochrane Prognosis Methods Group* [31] and *Cochrane Handbook for Systematic Reviews of Interventions* [32].

### 2.1. Protocol

In order to minimize risk of bias and improve the transparency, precision, and integrity of this systematic review and meta-analysis, a protocol on its methodology has been submitted *a priori* in *PROSPERO International prospective register of systematic reviews* (www.crd.york.ac.uk/PROSPERO) (accessed on 15 March 2021) (ID209729/CRD42020209729 code was assigned). The protocol followed complied with PRISMA-P statement to ensure a rigorous scientific approach [33].

### 2.2. Search Strategy

We searched PubMed, Embase, Web of Science, and Scopus databases for studies published before the search date (upper limit = May, 2020), with no lower date limit. Searches were built to maximize sensitivity and conducted by combining thesaurus terms used by the databases (i.e., MeSH and EMTREE) with free terms (Table S1, Supplementary Materials p. 3). 

An additional screening was performed handsearching the reference lists of retrieved included studies. All references were managed using Mendeley v.1.19.4 (Elsevier. Amsterdam, The Netherlands); duplicate references were removed using this software.

### 2.3. Eligibility Criteria

Inclusion criteria: Original studies—without language, publication date, follow-up periods, study design, geographical area, sex, or age restrictions—evaluating SP and/or NK-1R expression in human tissues from patients with tumors in the head and neck region. The names and affiliations of authors, the recruitment period, and setting were examined to determine whether studies were conducted in the same study population. In such cases, we included the most recent study or that which published more complete data.

Exclusion criteria were: Retractions, case reports, personal opinions or comments, meeting abstracts, books, bioinformatics analyses of microarray datasets, reviews or meta-analyses; in vitro or animal research; tumors or tissues from different anatomical areas; evaluation of SP and/or NK-1R gene alterations (e.g., polymorphisms); no analysis of the expression of these proteins or lack of data for their estimation with 95%CI. 

Study eligibility criteria were applied independently by two authors (MAGM and PRG). Any discrepancies were resolved by consensus; the agreement between reviewers on study eligibility was evaluated using Cohen’s kappa statistic, obtaining kappa (κ)-values. The articles were selected in two phases, first screening the titles and abstracts of retrieved articles (100% of agreement; κ = 1.00); second, reading the full text of the initial selection, excluding articles that did not meet the precedent eligibility criteria (95% of agreement; κ = 0.83).

### 2.4. Data Extraction

Two authors (MAGM and PRG) independently extracted data from the selected articles after full text reading, completing a data collection form in a standardized manner using Excel v.2015 (Microsoft. Redmond, WA), solving discrepancies by consensus (99.43% of agreement). Data were gathered on the first author, publication year, country and continent, publication language, biomarker under study (SP and/or NK-1R), sample size, tumor type, anatomical site and subsites affected, sex and age of patients, tobacco and alcohol consumption, treatment modality, recruitment and follow-up period, study design, methodology and the frequency of proteins expression. In immunohistochemical studies, information was also recorded on the anti-SP and/or NK-1R antibodies, intracellular immunostaining (nuclear/cytoplasmic/membrane), cutoff point, and scoring system. 

### 2.5. Evaluation of Quality and Risk of Bias

Two authors (MAGM and PRG) critically appraised the quality and risk of bias of studies using the *Quality in Prognosis Studies (QUIPS) tool* (Cochrane Prognosis Methods Group [34]). The development of this tool was based on an examination of numerous systematic reviews of prognostic studies [35], and six common areas of potential bias (*aka* domains) were identified [34]. Thus, in our studies sample, the following six main potential bias domains were explored: (1) Study participation; (2) Study attrition; (3) Prognostic factor measurement; (4) Outcome measurement; (5) Study confounding; (6) Statistical analysis/reporting. The risk of bias was evaluated as low, moderate, or high for each domain. Domains were independently evaluated in each individual study by both authors, who recorded the particularities and potential biases observed. Discrepancies were also resolved by consensus. The inter-agreement between the two authors (MAGM and PRG) was recorded, obtaining an agreement of scores for 89.58% across all items.

### 2.6. Statistical Analysis

SP and/or NK-1R expression was considered as high or low for a case in agreement with the methodology and cutoff values provided by the authors of each study. Proportions were calculated in individual studies extracting the raw numerators (cases with high expression for SP/NK-1R) and denominators (total cases). These proportions and their 95% confidence intervals (CIs) were meta-analyzed obtaining pooled proportions (PP) expressed as percentage. 95%CI were constructed based on the score-test statistic [36]. To minimize the influence of extremely small sample sizes, the variance of the study-specific prevalence was stabilized by Freeman-Tukey double arcsine transformation [37], computing the weighted pooled estimate and performing the back-transformation to pooled prevalence estimate [38]. All meta-analyses were conducted using a random-effects model (REM), based on the DerSimonian and Laird method (D-L), which accounts for the possibility that are different underlying results among study subpopulations (e.g., differences among tumors, head, and neck anatomical sites, or geographic areas). Forest plots were created to graphically represent the general effect and for its subsequent visual inspection analysis (*p* < 0.05 was considered significant). Heterogeneity between studies was checked applying the χ^2^-based Cochran’s Q test (given its low statistical power, *p* < 0.10 was considered significant) and quantified using Higgins I^2^ statistic (values of 50–75% were interpreted as moderate-to-high degree of inconsistency across the studies), which estimates what proportion of the variance in observed effects reflects variation in true effects, rather than sampling error [39,40].

As secondary analyses, we also conducted preplanned stratified meta-analyses (by malignant behavior of tumors, geographical area, anatomical site, histological and clinical type, and by biomarker) to identify potential sources of heterogeneity and to analyze the relationship of SP/NK-1R high expression among subgroups. Subgroups meta-analyses by anti-SP/NK-1R antibodies, cutoff point, and immunostaining patterns could not be carried out due to the substantial heterogeneity found between studies, covering a wide range of experimental methods or failing to report insufficient information. In addition, we conducted sensitivity analyses to test the reliability of meta-analytical results and to explore the influence of each individual study on the final estimations [41]. For this, the meta-analyses were repeated sequentially, omitting one study at a time (“leave-one-out” method). Finally, funnel plots were constructed, and the original Egger regression test for funnel plot asymmetry was applied to evaluate small-study effects, such as publication bias [42,43,44]. It was designed to perform a linear regression of the effect estimates on their standard errors, weighting by 1/(variance of the effect estimate); *p_Egger_* < 0.10 was considered significant. Stata version 16.1 (Stata Corporation, College Station, TX, USA) was employed for all tests, manually typing the commands syntax (PRG) [45].

## 3. Results

### 3.1. Literature Search

**Figure 1:**
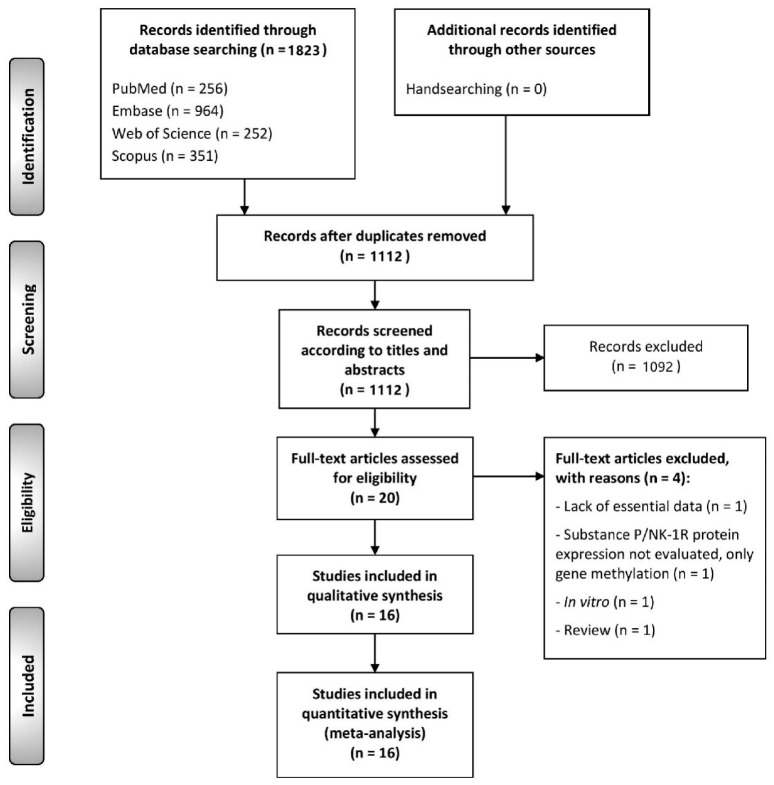
Flow diagram of the identification and selection of relevant studies, analyzing the differential expression of Substance P/NK-1R in head and neck tumorigenesis.

The flow diagram in Figure 1 depicts the study selection process and the results obtained. 1823 total publications were retrieved from PubMed (*n* = 256), Embase (*n* = 964), Web of Science (*n* = 252) and Scopus (*n* = 351). After eliminating duplicates, 1112 records were considered potentially eligible and their titles and abstracts were screened, leaving a sample of 20 studies for full text assessment. After excluding studies that did not meet all eligibility criteria (listed in the Supplementary Materials with exclusion reasons, p. 23), 16 studies were finally included in the systematic review for qualitative evaluation and quantitative meta-analysis (the references of the studies included were also listed in the Supplementary Materials, p. 24, 23).

### 3.2. Study Characteristics

Table S2 (Supplementary Materials, pp. 4, 5) exhibits in detail the variables gathered from each study, and Table 1 summarizes the characteristics of the 16 selected studies, which reported a total of 909 tumors from the head and neck region. Sample sizes ranged between 5 and 114 cases. SP expression was studied by 15 studies (897 cases) and NK-1R by 7 studies (411 cases); it should be noted that more than one biomarker was analyzed per study (Table S2). The anatomical sites involved were oral cavity (4 studies/285 cases), nasal cavity (1 study/20 cases), larynx (2 studies/233 cases), salivary glands (1 study/171 cases), thyroid gland (6 studies/142 cases) and head and neck mixed (2 studies/58 cases). Five studies (226 cases) assessed benign tumors, 3 studies (230 cases) pre-malignant tissues, and 11 studies (453 cases) malignant tumors. The studies were conducted in Europe (*n* = 11), Asia (*n* = 3) and North-Central America (*n* = 2). In relation to their study design, all were observational restrospective studies (*n* = 16). Cutoff points to measure SP/NK-1R expression were heterogeneous across studies. Occasionally, anti-SP/NK-1R antibodies and immunostaining patterns were not specified (Table S2).

### 3.3. Qualitative Evaluation

**Figure 2:**
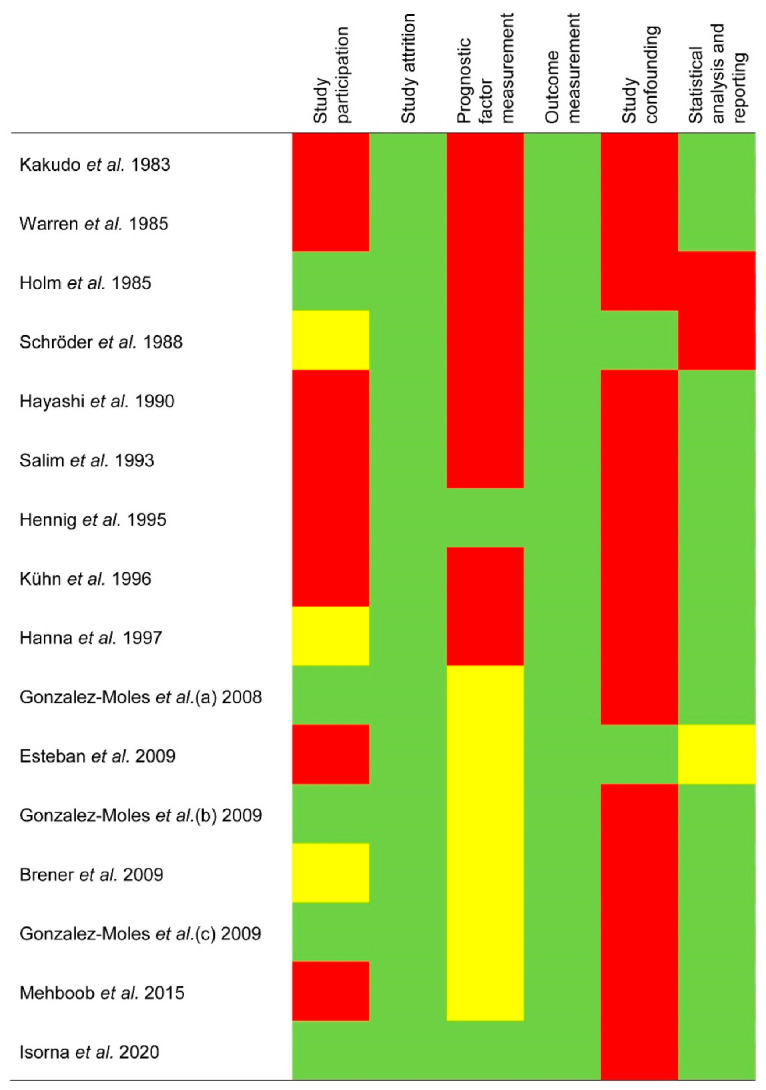
Evaluation of the risk of bias using the Quality in Prognosis Studies (QUIPS) tool.

The qualitative analysis was conducted using the QUIPS tool, which evaluates potential sources of bias in six domains (Figure 2):

Study participation. The risk of this bias was high in 50% of the reviewed studies, moderate in 18.75%, and low in 31.25% (Figure 2). The potential biases were the inadequate description of the main patient characteristics: sex, age, anatomical subsites, and habits (i.e., tobacco and/or alcohol consumption).

Study attrition. All studies (100%) showed a low risk of potential bias (Figure 2). The failure to report on patients lost to the follow-up or an inadecuate description of this period did not exert an impact on SP/NK-1R differential expression ratios. 

Prognostic factor measurement. The bias risk was high in 50% of the studies, moderate in 37.50%, and low in 12.50% (Figure 2). The most frequent biases included insufficient information on the immunohistochemical technique (e.g., antibody clones) and on the scoring systems (e.g., methods used for cutoff points determination). More important limitations such as the application of inappropriate cutoff points (e.g., use of optimized cutoff points based on data analysis, which can introduce strong biases [46]) or the failure to measure SP/NK-1R in a similar way for all cases were not found.

Outcome measurement. All studies (100%) showed a low risk of potential bias (Figure 2). The biases related to the failure reporting clinicopathological (e.g., lack of information on TNM staging edition or methods) or survival variables (e.g., non-definition of endpoints) had no impact on the differential expression ratios of SP/NK-1R.

Study confounding. The bias risk was high in 87.50% of the studies, and low in 12.50% (Figure 2), finding the lowest potential bias in this domain. The most frequent potential biases were the failure to consider confounders in the study design. Although in some studies, multivariable analyses were performed adjusting for potential confounders, no study provided a priori clear definitions of these factors nor subsequently discussed the biological mechanisms by which they might impact SP/NK-1R overexpression.

Statistical analysis and reporting. The risk of this bias was high in 12.50% of the studies, moderate in 6.25%, and low in 81.25% (Figure 2), most frequently due to an inappropriate statistical analysis or the identification of potential selective reporting.

### 3.4. Quantitative Evaluation (Meta-Analysis)

#### 3.4.1. Differential Expression of SP/NK-1R in Head & Neck Tumorigenesis

**Figure 3:**
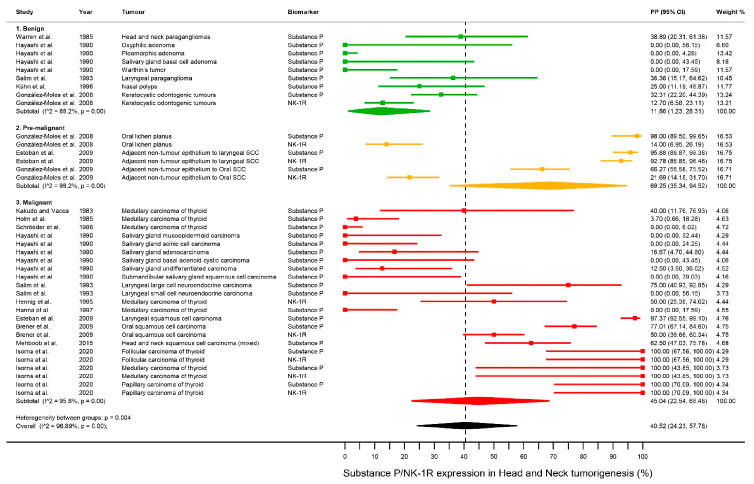
Forest plot. A forest plot graphically representing the stratified meta-analysis on differential expression of SP/NK-1R in head & neck tumorigenesis by malignant behavior. ES, effect size (i.e., pooled proportions expressed as percentage); CI, confidence intervals. Random-effects model, inverse-variance weighting (based on the DerSimonian and Laird method).

SP/NK-1R showed a high expression in a relevant proportion of cases in head and neck tumorigenesis (benign and malignant tumors and pre-malignant tissues) (PP = 40.52%, 95%CI = 24.23–57.78) (Figure 3, Table 2), although a considerable degree of heterogeneity was present (*p* < 0.001, I^2^ = 96.89%), indicating that all tumors do not display similar expression levels. After the subsequent analysis stratifying tumors by malignant behavior, the highest proportion of cases with high expression were found in malignant tumors (PP = 45.04% [95%CI = 22.54–68.48]) and pre-malignant tissues (PP = 69.25%, 95%CI = 35.34–94.52), and the lowest in benign tumors (PP = 11.86%, 95%CI = 1.23–28.31), with significant differences between subgroups (*p* = 0.004). The pre-malignant and malignant groups were also combined, obtaining a high expression for SP/NK-1R, approximately one half of the cases (PP = 50.93%, 95%CI = 32.13–69.61), also showing significant differences with the benign tumors group (*p* = 0.002). Furthermore, paired analyses were additionally performed, confirming once again the precedent results, and also showing non-significance differences between the malignant and pre-malignant groups (*p* = 0.24) (Table 2, Figures S1–S4, Supplementary Materials pp. 6–9).

#### 3.4.2. Differential Expression of SP/NK-1R in Benign Tumors

Significant differences for SP/NK-1R expression were found after the stratification of benign tumors by geographical area (Asia: PP = 0.00% (95%CI = 0.00–0.00); Non-Asia: PP = 26.43% (95%CI = 15.41–39.01); *p* < 0.001), anatomical sites (larynx: PP = 36.36% [95%CI = 15.17–64.62], nasal cavity: PP = 25.00% [95%CI = 11.19–46.87], oral cavity: PP = 21.88% [95%CI = 15.04–29.56], salivary glands: PP = 0.00% [95%CI = 0.00–0.00] and head and neck mixed: PP = 38.89% [95%CI = 20.31–61.38]; *p* < 0.001) and histological types (Glandular: PP = 0.00% [95%CI = 0.00–0.00]; squamous: PP = 24.17% [95%CI = 12.56–37.86]; nervous: PP = 38.89% [95%CI = 20.31–61.38]; *p* < 0.001), but not between biomarkers (SP: PP = 11.77% [95%CI = 0.11–32.64]; NK-1R: PP = 12.70% [95%CI = 6.58–23.11]; *p* = 0.85) (Table 2, Figures S5–S8, Supplementary Materials pp. 10–13).

#### 3.4.3. Differential Expression of SP/NK-1R in Pre-Malignant Tissues

Significant differences for SP/NK-1R expresion were observed after the stratification of pre-malignant tissues by anatomical sites (Larynx: PP = 94.42% [95%CI = 90.63–97.34]; oral cavity: PP = 52.24% [95%CI = 13.57–89.40], *p* = 0.02) and clinical types (oral lichen planus: PP = 61.30% [95%CI = 51.45–70.72]; adjacent non tumor epithelium to oral squamous cell carcinoma: PP = 43.27% [95%CI = 35.78–50.93]; adyacent non tumor epithelium to larynx squamous cell carcinoma: 94.42% [95% = 90.63–97.34]; *p* < 0.001). However, no significant difference (*p* = 0.13) was observed in the proportion of cases with high expression between SP (PP = 89.60% [95%CI = 66.10–100.00]) and NK-1R (PP = 43.92% [95%CI = 1.33–93.97]) (Table 2, Figures S9–S11, Supplementary Materials pp. 14–16). 

#### 3.4.4. Differential Expression of SP/NK-1R in Pre-Malignant Tissues

Significant differences for SP/NK-1R expression were found after the stratification of malignant tumors by geographical area (Asia: PP = 12.04% [95%CI = 0.00–35.87); Non-Asia: PP = 65.03% [95%CI = 34.63–90.69]; *p* = 0.007), anatomical site (larynx: PP = 67.80% [95%CI = 8.94–100.00]; oral cavity: PP = 64.14% [95%CI = 56.80–71.17]; salivary gland: PP = 3.84% [95%CI = 0.00–12.24], thyroid gland: PP = 63.25% [95%CI = 22.83–96.04]; Head and neck mixed: 62.50% [95%CI = 47.03–75.78]; *p* < 0.001), and biomarkers (SP: PP = 33.60% [95%CI = 9.13–62.90]; NK-1R (PP = 83.67% [95%CI = 51.40–100.00]; *p* = 0.02) (Table 2, Figure S12–15, Supplementary Materials pp. 17–10).

### 3.5. Quantitative Evaluation (Secondary Analyses)

#### 3.5.1. Sensitivity Analysis

In general, the pooled results did not substantially change after the sequential repetition of meta-analyses, omitting one study each turn (Tables S3–S5, Supplementary Materials pp. 21, 22). The most stable results were those reported for the series of malignant tumors, due to the greater number of observations under analysis. Sensitivity analyses suggest that the combined estimations reported do not depend on the influence of a particular individual study.

#### 3.5.2. Analysis of Small-Study Effects

**Figure 4:**
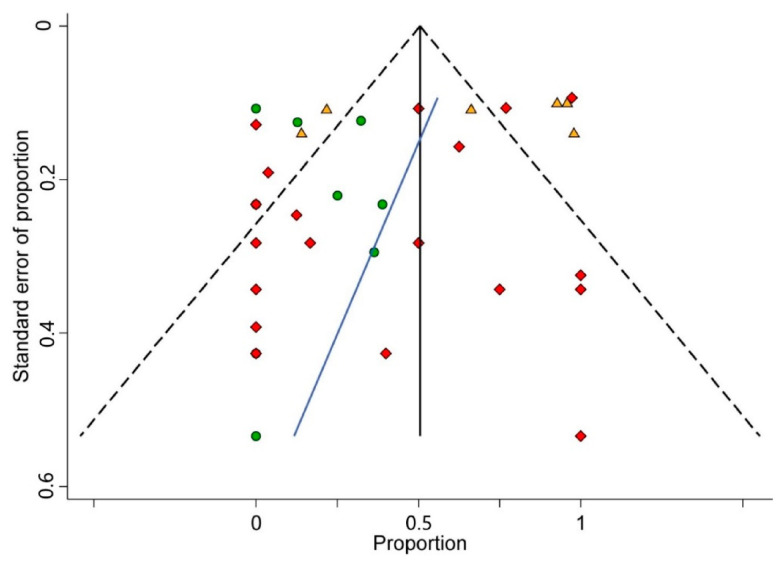
Funnel plot. A funnel plot of estimated proportion of cases with high expression for SP/NK-1R against its standard error in head and neck tumorigenesis. The black vertical line corresponds to the pooled proportion of cases with high expression for SP/NK-1R. The two diagonal intermittent lines represent the pseudo-95% confidence interval. The green circles, orange triangles, and red diamonds represent the published series reporting SP/NK-1R expression in benign, pre-malignant, and malignant tumors, respectively. The blue line represents the fitted line corresponding to Egger’s regression test (*p* = 0.190) for funnel plot asymmetry.

Visual inspection analysis of the asymmetry of the funnel plot constructed (Figure 4) and the statistical test conducted for the same purpose (*p_Egger_* = 0.190) confirmed the absence of small-study effects. Therefore, publication bias could be potentially ruled out.

### 3.6. Biological and Oncogenic Roles of SP/NK-1R

Due to the low number of observations derived from our sample focusing on the relationship between SP/NK-1R with other biomarkers, the biological and oncogenic roles of SP/NK-1R could not be tested using meta-regression or other meta-analytical techniques. Nevertheless, from a narrative synthesis point of view, three of the studies included in this systematic review have shown that tissues with high expression of SP/NK-1R also showed Ki-67 overexpression in oral squamous cell carcinomas, oral lichen planus, and keratocystic odontogenic [11,28,47]; therefore, uncontrolled cell proliferation could be the putative oncogenic role of SP/NK-1R in pre-malignant and malignant cells, potentially activating downstream central oncogenic signaling pathways (i.e., MAPK and PI3K [15,16,17,18,19]). Furthermore, future studies are necessary to investigate whether the emerging oncogenic functions of SP/NK-1R also have implications in head and neck oncogenesis (e.g., increased cell migration, invasiveness and metastasis, neoangiogenesis, and chronic inflammation, cell death evasion, and reprogramming energy metabolism).

Finally, the overexpression of SP/NK-1R and their roles regulating these relevant signaling molecular networks also justifies the opportunistic interest of investigating the potential usefulness of SP/NK-1R as a therapeutic target in cancer. In this regard, interesting NK-1R antagonists (e.g., aprepitant) have shown promising value as antitumor drugs in other locations and should also be tested in head and neck tumors (see discussion).

## 4. Discussion

The results of this systematic review and meta-analysis show that jointly considered SP/NK-1R overexpression is significantly higher in pre-malignant and malignant head and neck lesions compared to benign lesions (*p* = 0.002). Subgroup analysis also shows that there are no SP/NK-1R overexpression significant differences between pre-malignant and malignant head and neck lesions (*p* = 0.24). The scientific evidence provided in relation to these results of our meta-analysis shows that the oncogenic actions linked to SP/NK-1R are relevant in head and neck oncogenesis behaving as an early event. Furthermore, three of the studies included in this meta-analysis have shown, in oral squamous cell carcinomas, oral lichen planus and keratocystic odontogenic, that tissues with overexpression of SP/NK-1R also showed a high expression of Ki-67 [11,28,47]; similar findings have also been reported through Ki-67 labeling index assessment in other human cancers, such as Hepatoblastoma [48] or leukemia [49]. It seems to indicate that probably the constitutive activation of the receptor in pre-malignant and malignant cells behaves as a regulatory mechanism of oncogenic signaling pathways that induce proliferation, especially MAPK and PI3K [15,16,17,18,19]. Today it is accepted that the increase in proliferative activity is the most relevant hallmark of cancer, as a consequence of the genomic instability that it generates and the associated risk of acquiring summative oncogenic events that will eventually lead to malignant transformation. In addition, our meta-analysis also indicates that, in malignant tissue, there is a greater up-regulation of NK-1R compared to SP (*p* = 0.02), so it could be hypothesized that once the epithelial cell has transformed, the maintenance of a clonal state of upregulation of the receptor will mediate the gain of oncogenic advantages with effects on migration, metastasis, etc. Future studies are necessary to corroborate whether the emerging oncogenic functions of SP/NK-1R—e.g., cell migration gain and invasiveness, neoangiogenesis and chronic inflammation-promotion, or resistance to apoptosis—could also play key roles in head and neck oncogenesis, as has been demonstrated in other human cancers (e.g., endometrial adenocarcinoma, breast or pancreatic cancer) [20,21,22,23,24,25]. Likewise, the evidence provided in this meta-analysis highlights the interest in the development of in-depth research on targeted therapies directly to the receptor, such as L-773060, a specific NK-1R antagonist that has demonstrated its antitumor activity against different cell lines (gliomas, retinoblastoma, neuroblastoma, melanoma or laryngeal carcinoma) [15,17,18,50,51,52] and aprepitant that has already been tested in humans [25,26,53,54,55].

The overexpression of SP and NK-1R has been presented differentially in geographical areas of the world, being more infrequent in Asia compared to the rest of the world for both benign (*p* < 0.001) and for malignant lesions (*p* = 0.007). This comparison has not been possible to be performed in pre-malignant epithelia since no studies have been published in this regard in Asia. This is the first time that a result has been presented in head and neck carcinogenesis in this sense, for SP and NK-1R, although at least for oral cancer, a geographically different expression has been reported for other important oncogenes, i.e., the RAS family [56]. The mechanisms justifying this difference are unknown, although in relation to the etiology of head and neck cancer, particularly oral cancer, the key difference between Asia and the rest of the world is that Asia is the geographical region with the highest rate of global tobacco use [57]. There is very little research regarding the effects of tobacco use on the expression of SP and NK-1R, although an experimental study has shown inhibition of NK-1R-mediated signaling in response to second-hand tobacco smoke exposure [58].

Our results also show that there is a differential expression related to the affected organ in the head and neck, both in benign and in malignant lesions, observing a null or very infrequent overexpression in lesions of the salivary glands compared to that found in malignant squamous tissue, both in the larynx and in the oral cavity (*p* < 0.001), which reveals the scarce importance of the pathways activated by SP/NK-1R in glandular neoplasms of the head and neck.

According to our qualitative evaluation using QUIPS tool, although the studies in our meta-analysis had similar study design—in experimental and epidemiological terms—all were not conducted with the same scientific rigor. Most potential biases were caused by the failure to consider potentially confounding factors (study confounding domain), and due to insufficient reporting on the biomarkers under investigation (prognostic factor measurement domain). Singularly, studies should more meticulously communicate details on the immunohistochemical methods (e.g., antibody clones, methods used for cutoff points determination, scoring systems, etc.). Future studies assessing the relationships between SP/NK-1R among subjects with head and neck tumors could consider the recommendations given in this systematic review and meta-analysis to improve and standardize future research.

Some potential limitations should also be discussed. First, our meta-analysis revealed a considerable heterogeneity degree. Heterogeneity is a common finding in meta-analyses dealing with proportions [59,60], and it must be noted that a random-effects model was applied to account for heterogeneity. Furthermore, after several stratification analyses that allowed for more homogenous inter-subgroup distributions, potential sources of heterogeneity may have been identified (e.g., differential expression of SP/NK-1R and malignant behavior of tumors, geographical regions, anatomical area and histological type). An additional potential source of methodological heterogeneity was observed according to immunohistochemical technique, particularly in relation to its application, anti-SP/NK-1R antibodies and cutoff points used to identify a case as positive. Thirteen studies did not report the clone and/or dilution used, and no studies reported the mono/polyclonal nature of the antibody. Although the majority of groups reported the cutoff points, the observed variability—1%/13 studies, 10%/1 study, 30%/1 study, not reported/1 study—made it complex to issue recommendations based on a high quality of evidence. Finally, due to a lack of published data, meta-analyses on clinicopathological variables and/or prognostic variables (e.g., time to event variables as overall, diseases-specific, or disease-free survival) could not be performed. Future studies investigating the prognostic value of SP/NK-1R in head and neck cancer are needed, encouraged by the interest and opportunities observed and discussed in this meta-analysis. Despite the above limitations, study strengths include our careful study design, a sensitive literature search strategy enhanced by the absence of restrictions by publication language or date limits, and robust qualitative recommendations for future studies on this topic. Finally, our meta-analytical results are reliable and stable, as respectively supported by the small-study effects and sensitivity analyses.

## 5. Conclusions

In conclusion, the results of this systematic review and meta-analysis show evidence that the upregulation of SP and NK-1R are oncogenic events involved in head and neck carcinogenesis, probably acting in the early stages of malignization. In addition, there is evidence of a greater relevance of the upregulation of the NK-1R receptor compared to SP. It highlights the interest in deepening the development of targeted therapies on the receptor in head and neck oncogenesis, testing inhibitors such as aprepitant, which has already shown translational potential in several human cancers (e.g., breast, colon, gastric, laryngeal, lung, pancreatic cancers, esophageal squamous cell carcinoma, or melanoma).

## Figures and Tables

**Table 1:** Summarizes the main characteristics of reviewed studies. Table S2 (in Supplementary Materials, pp. 4, 5) exhibits in detail the characteristics of each study. *—More than one biomarker and type of tumor were analyzed per study.

Total	16 Studies
Year of publication	1983–2020
Number of cases	
Total	909
Sample size, range	5–114
Biomarkers analyzed *	
SP	15 studies (897 cases)
NK-1R	7 studies (411 cases)
Affected sites	
Oral cavity	4 studies (285 cases)
Nasal cavity	1 study (20 cases)
Larynx	2 studies (233 cases)
Salivary glands	1 study (171 cases)
Thyroid gland	6 studies (142 cases)
Head and neck mixed	2 studies (58 cases)
Type of tumors *	
Benign	5 studies (226 cases)
Pre-malignant	3 studies (230 cases)
Malignant	11 studies (453 cases)
Study design	
Retrospective cohort	16 studies
Geographical region	
Europe	11 studies (610 patients)
Asia	3 studies (216 cases)
North-Central America	2 studies (83 cases)

**Table 2:** Meta-analyses on differential expression of substance P (SP)/NK-1R in head & neck tumorigenesis and associated factors.

					Pooled Data		Heterogeneity		
Meta-Analyses	No. of Studies *	No. of Tumors	Stat. Model	Wt	PP (95% CI)	*p*-Value	*P_het_*	I^2^ (%)	Supplementary Materials ^a^
**1. All tumors**									
All ^b^	38	1308	REM	D-L	40.52% (24.23–57.78)	──	<0.001	96.89	Figure 3
Classification of tumors by malignant behavior ^c^						0.004 ^d^			Figure 3
Benign	9	289	REM	D-L	11.86% (1.23–28.31)		<0.001	88.2	
Pre-malignant	6	460	REM	D-L	69.25% (35.34–94.52)		<0.001	98.2	
Malignant	23	559	REM	D-L	45.04% (22.54, 68.48)		<0.001	95.8	
Classification of tumors by malignant behavior —combining the pre-malignant and malignant groups- ^c^						0.002 ^d^			Figure S1, p. 6
Benign	9	289	REM	D-L	11.86% (1.23–28.31)		<0.001	88.2	
Non-Benign	29	1019	REM	D-L	50.93% (32.13–69.61)		<0.001	96.6	
Paired analysis by malignant behavior						0.003			Figure S2, p. 7
Benign	9	289	REM	D-L	11.86% (1.23–28.31)		<0.001	88.2	
Pre-malignant	6	460	REM	D-L	69.25% (35.34–94.52)		<0.001	98.2	
Paired analysis by malignant behavior						0.02			Figure S3, p. 8
Benign	9	289	REM	D-L	11.86% (1.23–28.31)		<0.001	88.2	
Malignant	23	559	REM	D-L	45.04% (22.54, 68.48)		<0.001	95.8	
Paired analysis by malignant behavior						0.24			Figure S4, p. 9
Pre-malignant	6	460	REM	D-L	69.25% (35.34–94.52)		<0.001	98.2	
Malignant	23	559	REM	D-L	45.04% (22.54, 68.48)		<0.001	95.8	
**2. Benign tumors**									
Benign tumors by geographical area ^c^						<0.001 ^d^			Figure S5, p. 10
Asian	4	112	REM	D-L	0.00% (0.00–0.00)		0.74	0.0	
Non-Asian	5	177	REM	D-L	26.43% (15.41–39.01)		0.04	60.96	
Benign tumors by anatomical site ^c^						<0.001 ^d^			Figure S6, p. 11
Larynx	1	11	──	──	36.36% (15.17–64.62)		──	──	
Nasal cavity	1	20	──	──	25.00% (11.19–46.87)		──	──	
Oral cavity	2	128	REM	D-L	21.88% (15.04–29.56)		──	──	
Salivary gland	4	112	REM	D-L	0.00% (0.00–0.00)		0.76	0.0	
Head and neck mixed	1	18	──	──	38.89% (20.31–61.38)		──	──	
Benign tumors by histological type ^c^						<0.001 ^d^			Figure S7, p. 12
Glandular	4	112	REM	D-L	0.00% (0.00–0.00)		0.76	0.0	
Squamous	4	159	REM	D-L	24.17% (12.56–37.86)		0.04	64.01	
Nervous	1	18	──	──	38.89% (20.31–61.38)		──	──	
Benign tumors by biomarker ^c^						0.85 ^d^			Figure S8, p. 13
SP	8	226	REM	D-L	11.77% (0.11–32.64)		<0.001	89.64	
NK-1R	1	63	──	──	12.70% (6.58–23.11)		──	──	
**3. Pre-malignant tissues**									
Pre-malignant tissues by geographical area ^c^						──			──
Asian	0	0	──	──	──		──	──	
Non-Asian	6	460	REM	D-L	69.25% (35.34–94.52)		<0.001	98.2	
Pre-malignant tissues by anatomical site ^c^						0.02 ^d^			Figure S9, p. 14
Larynx	2	194	REM	D-L	94.42% (90.63–97.34)		──	──	
Oral cavity	4	266	REM	D-L	52.24% (13.57–89.40)		0.001	97.94	
Pre-malignant tissues by histological type ^c^						──			──
Squamous	6	460	REM	D-L	69.25% (35.34–94.52)		<0.001	98.2	
Other	0	0	──	──	──		──	──	
Pre-malignant tissues by clinical type ^c^						<0.001 ^d^			Figure S10, p. 15
OLP	2	100	REM	D-L	61.30% (51.45–70.72)		──	──	
ANTE-OSCC	2	166	REM	D-L	43.27% (35.78–50.93)		──	──	
ANTE-LSCC	2	194	REM	D-L	94.42% (90.63–97.34)		──	──	
Pre-malignant tissues by biomarker ^c^						0.13 ^d^			Figure S11, p. 16
SP	3	230	REM	D-L	89.60% (66.10–100.00)		<0.001	94.77	
NK-1R	3	230	REM	D-L	43.92% (1.33–93.97)		<0.001	98.75	
**4. Malignant tumors**									
Malignant tumors by geographical area ^c^						0.007 ^d^			Figure S12, p. 17
Asian	8	104	REM	D-L	12.04% (0.00–35.87)		<0.001	83.55	
Non-Asian	15	455	REM	D-L	65.03% (34.63–90.69)		<0.001	96.81	
Malignant tumors by anatomical site ^c^						<0.001 ^d^			Figure S13, p. 18
Larynx	3	125	REM	D-L	67.80% (8.94–100.00)		<0.001	90.58	
Oral cavity	2	173	REM	D-L	64.14% (56.80–71.17)		──	──	
Salivary gland	6	59	REM	D-L	3.84% (0.00–12.24)		0.55	0.0	
Thyroid gland	11	162	REM	D-L	63.25% (22.83–96.04)		<0.001	95.15	
Head and neck mixed	1	4	──	──	62.50% (47.03–75.78)		──	──	
Malignant tumors by histological type ^c^						0.16 ^d^			Figure S14, p. 19
Glandular	17	221	REM	D-L	37.31% (12.70–65.25)		<0.001	92.71	
Squamous	4	327	REM	D-L	74.83% (47.01–94.77)		<0.001	96.27	
Neuroendocrine	2	11	REM	D-L	51.42% (19.04–83.29)		──	──	
Malignant tumors by biomarker ^c^						0.02 ^d^			Figure S15, p. 20
SP	18	441	REM	D-L	33.60% (9.13–62.90)		<0.001	96.57	
NK-1R	5	118	REM	D-L	83.67% (51.40–100.00)		<0.001	84.57	

Abbreviations: Stat., statistical; Wt, method of weighting; PP, pooled proportion; CI, confidence intervals; REM, random-effects model; D-L, DerSimonian and Laird method; OLP, oral lichen planus; ANTE-OSCC, adjacent non-tumor epithelium to oral squamous cell carcinoma; ANTE-LSCC, djacent non-tumor epithelium to laryngeal squamous cell carcinoma. * More than one type of tumor was analyzed per study and considered separately. a—More information in the Supplementary Materials; b—Proportion meta-analyses; c—Proportion meta-analyses (Subgroup analyses); d—Test for between-subgroup differences.

## Data Availability

The data that supports the findings of this study are available in the Supplementary material of this article.

## Supplementary Materials

The following are available online at https://www.mdpi.com/2072-6694/13/6/1349/s1, Figures S1–S4: tumorigenesis by malignant behavior, Figure S5: benign tumours by geographical area, Figure S6: benign tumours by anatomical site, Figure S7: benign tumours by histological type, Figure S8: benign tumours by biomarker, Figure S9: pre-malignant tissues by anatomical site, Figure S10: pre-malignant tissues by clinical type, Figure S11: pre-malignant tissues by biomarker, Figure S12: malignant tumours by geographical area, Figure S13: malignant tumours by anatomical site, Figure S14: malignant tumours by histological type, Figure S15: malignant tumours by biomarker, Table S1: Search strategy for each database, number of results, and execution date, Table S2: Characteristics of the analyzed studies, Table S3: Sensitivity analysis of the studies pooled in the meta-analysis on the expression of substance P/NK-1R in head and neck benign tumours, Table S4: Sensitivity analysis of the studies pooled in the meta-analysis on the expression of substance P/NK-1R in head and neck pre-malignant tissues, Table S5: Sensitivity analysis of the studies pooled in the meta-analysis on the expression of substance P/NK-1Rin head and neck malignant tumours
