# Supplementary material for: Significance of the Overexpression of Substance P and Its Receptor NK-1R in Head and Neck Carcinogenesis: A Systematic Review and Meta-Analysis
Source: Cancers (Basel). 2021 Mar 17;13(6):1349. doi: 10.3390/cancers13061349 (PMC8002440; doi:10.3390/cancers13061349)
Supplement: Supplementary file 1 [file cancers-13-01349-s001.pdf]

Appendix to the manuscript

Significance of the overexpression of substance P and its receptor NK-1R in head and neck carcinogenesis: a systematic review and meta-analysis

Miguel Ángel González-Moles [1, 2, 3,*], Pablo Ramos-García [1, 2, *] and Francisco Esteban [4]

[1] School of Dentistry, University of Granada, Granada, Spain
[2] Instituto de Investigación Biosanitaria ibs.GRANADA, Granada, Spain
[3] WHO Collaborating Group for Oral Cancer
[4] Department of Otorhinolaryngology, Virgen del Rocio University Hospital, Sevilla, Spain

* Correspondence: magonzal@ugr.es (MAGM), pramos@correo.ugr.es (PRG).

Table of contents

1. Search strategy. Table S1. 3

2. Characteristics of analyzed studies. Table S2 4

3. Subgroup meta-analyses on differential expression of substance P/NK-1R in head & neck tumorigenesis and associated factors 6

Fig.S1 tumorigenesis by malignant behavior (benign vs. non-benign) 6

Fig.S2 tumorigenesis by malignant behavior (benign vs. pre-malignant) 7

Fig.S3 tumorigenesis by malignant behavior (benign vs. malignant)......................8

Fig.S4 tumorigenesis by malignant behavior (pre-malignant vs. malignant) 9

Fig.S5 benign tumours by geographical area 10

Fig.S6 benign tumours by anatomical site 11

Fig.S7 benign tumours by histological type..12

Fig.S8 benign tumours by biomarker..13

Fig.S9 pre-malignant tissues by anatomical site 14

Fig.S10 pre-malignant tissues by clinical type 15

Fig.S11 pre-malignant tissues by biomarker...16

Fig.S12 malignant tumours by geographical area 17

Fig.S13 malignant tumours by anatomical site 18

Fig.S14 malignant tumours by histological type 19

Fig.S15 malignant tumours by biomarker...20

4. Sensitivity analysis 21

4.1 Differential expression of substance P/NK-1R in benign tumours..................21

4.2 Differential expression of substance P/NK-1R in pre-malignant tissues.........21

4.3 Differential expression of substance P/NK-1R in malignant tumours.............22

15. List of full-text articles excluded with reasons...23

14. List of studies included in this systematic review and meta-analysis. List S5..........24

1. Search strategy

Table S1. Search strategy for each database, number of results, and execution date.

Database	Query	Results	Upper limit
PubMed	(“Substance P”[mh] OR “substance p”[all] OR “p substance”[all]) OR (“Receptors, Neurokinin-1”[mh] OR “neurokinin 1 receptor”[all] OR “tachykinin receptor 1”[all] OR “substance P receptor”[all] OR “NK1R”[all] OR “NK 1R”[all] OR (“NK 1”[all] AND “receptor”[all]) OR “TACR1”[all]) AND (“head”[mh] OR “head”[all] OR “neck”[mh] OR “neck”[all] OR "mouth"[mh] OR "mouth"[all] OR "oral"[all] OR "pharynx”[mh] OR pharyn*[all] OR oropharyn*[all] OR nasopharyn*[all] OR hypopharyn*[all] OR "larynx”[mh] OR laryn*[all] OR "nose”[mh] OR “nose”[all] OR “nasal”[all] OR “Salivary Glands”[mh] OR “salivary”[all] OR “Thyroid Gland”[mh] OR “thyroid”[all]) AND ("Head and Neck Neoplasms"[mh] OR neoplas*[all] OR “tumor”[all] OR “tumour”[all] OR benign*[all] OR "carcinoma, squamous cell"[mh] OR "carcinoma"[all] OR “cancer”[all] OR malign*[all])	256	May, 2020
Embase	('substance P'/exp OR 'substance' NEAR/2 'p') OR ('neurokinin 1 receptor'/exp OR 'neurokinin 1 receptor' OR 'tachykinin receptor 1' OR 'substance P receptor' OR 'NK1R' OR 'NK 1R' OR ('NK 1' AND 'receptor') OR 'TACR1') AND ('head'/exp OR 'head' OR 'neck'/exp OR 'neck' OR 'mouth'/exp OR 'mouth' OR 'oral' OR 'pharynx'/exp OR 'pharyn*' OR 'oropharyn*' OR 'nasopharyn*' OR 'hypopharyn*' OR 'larynx'/exp OR 'laryn*' OR 'nose'/exp OR 'nose' OR 'nasal' OR 'salivary gland'/exp OR 'salivary' OR 'thyroid gland'/exp OR 'thyroid') AND ('neoplasm'/exp OR 'neoplas*' OR 'benign head and neck tumor'/exp OR 'benign*' OR 'tumo$r' OR 'malignant neoplasm'/exp OR 'head and neck cancer'/exp OR 'squamous cell carcinoma'/exp OR 'carcinoma' OR 'cancer' OR 'malign*')	964	May, 2020
Web of Science	TS=(“substance p” OR “p substance” OR “neurokinin 1 receptor” OR “tachykinin receptor 1” OR “substance P receptor” OR NK1R OR “NK 1R” OR (“NK 1” AND receptor) OR TACR1) AND TS=(head OR neck OR mouth OR oral OR pharyn* OR oropharyn* OR nasopharyn* OR hypopharyn* OR laryn* OR nose OR nasal OR “salivary glands” OR “thyroid Gland”) AND TS=(neoplas* OR tumor OR tumour OR benign* OR carcinoma OR cancer OR malign*)	252	May, 2020
Scopus	TITLE-ABS-KEY((“substance p” OR “p substance” OR “neurokinin 1 receptor” OR “tachykinin receptor 1” OR “substance P receptor” OR “NK1R” OR “NK 1R” OR (“NK 1” AND “receptor”) OR “TACR1”) AND (“head” OR “neck” OR “mouth” OR “oral” OR “pharyn*” OR “oropharyn*” OR “nasopharyn*” OR “hypopharyn*” OR “laryn*” OR “nose” OR “nasal” OR “salivary glands” OR “thyroid Gland”) AND (“neoplas*” OR “tumor” OR “tumour” OR “benign*” OR “carcinoma” OR “cancer” OR “malign*”))AND (“squamous cell carcinoma” OR “neoplas*” or “cancer”))	351	May, 2020
Total			1,823

Table S2. Characteristics of the analyzed studies (n=16)

Study	Year	Country	Language	Biomarker analyzed	Tumor type (n)	Tumor site (n, subsites)	Sex, M/F	Age. years	Tobacco	Alcohol	recruitment period	therapy	Follow-up, months	Study design	Funding	Conflict of interest	Methods	IHC antibody	IHC pattern	IHQ Cutoff, %	Positivity %
Kakudo et al.	1983	Japan USA	English	Substance P	Medullary carcinoma of thyroid (5)	Thyroid gland (5)	NR	NR	NR	NR	NR	Sx	NR	O,R	NR	NR	IHQ	Clone NR, 1:100 to 1:2000	Intrafollicular	1	40
Warren et al.	1985	USA	English	Substance P	Paragangliomas (5)	Head and neck (18)	NR	NR	NR	NR	1973-1983	Sx	Range: 5-120	O,R,L	NR	NR	IHQ	Clone NR, 1:40	NR	1	38.89
Holm et al.	1985	Norway	English	Substance P	Medullary carcinoma of thyroid (27)	Thyroid gland (27)	12/15	Mean:47 Range: 14-75	NR	NR	1973-1983	Sx,Ct,Rt	<120	O,R,L	NR	NR	IHQ	Clone NR, 1:100	NR	1	3.70
Schröder et al.	1988	Germany	English	Substance P	Medullary carcinoma of thyroid (60)	Thyroid gland (60)	20/25 Missing:15	Mean:55 Range:18-81	NR	NR	NR	Sx	1.2-256.8	O,R,L	NR	NR	IHQ	Clone NR, 1:500	NR	1	0
Hayashi et al.	1990	Japan	English	Substance P	adenocarcinoma (12), undifferentiated carcinoma (16), acinic cell carcinoma (12),basal adenoid cystic carcinoma (5),Submandibular salivary gland squamous cell carcinoma (6),mucoepidermoid carcinoma (8),Pleomorphic adenoma (86),basal cell adenoma (5),Warthin's tumor (18), Oxyphilic adenoma (3)	Salivary glands (171)	NR	NR	NR	NR	NR	Sx	NR	O,R	NR	NR	IHQ	Clone NR, 1:200	NR	1	2.34
Salim et al.	1993	Uk	English	Substance P	Small cell neuroendocrine carcinoma (3), Large cell neuroendocrine carcinoma (8),Paraganglioma (11)	Larynx (22)	17/5	Range:33-80	NR	NR	NR	Sx	NR	O,R	NR	NR	IHQ	NR	NR	1	45.45
Hennig et al.	1995	Switzerland	English	NK-1R	Medullary carcinoma of thyroid (12)	Thyroid gland (12)	NR	NR	NR	NR	NR	Sx	NR	O,R	NR	NR	Autoradiography	–	NR	—	50.0
Kühn et al.	1996	Germany	English	Substance P	Nasal polyps (20)	Nasal cavity (20)	NR	Range:28-63	NR	NR	NR	Sx	0	O,R	NR	NR	IHQ	Clone NR, 1:100	NR	1	25.0
Hanna et al.	1997	Uk	English	Substance P	Medullary carcinoma of thyroid (18)	Thyroid gland (18)	14/9	NR	NR	NR	1973-1993	Sx	>180	O,R,L	NR	NR	IHQ	Clone NR, 1:200	NR	1	0.0
Gonzalez-Moles et al. (a)	2008	Spain	English	Substance P NK-1R	Keratocystic odontogenic tumours (65)	Oral cavity (posterior maxilla 12, posterior mandible 36,	37/28	Mean:27	NR	NR	NR	Sx	NR	O,R	NR	None	IHQ	Clone NR, 1:500	Substance P: Mixed membrane nuclear cytoplasmic	30	Substance P: 32.30 NK-1R: 12.70

						anterior mandible 4, body of mandible 5, anterior maxilla 8)													NK-1R: Mixed membrane cytoplasmic		(missing data: 2 cases)
Esteban et al.	2009	Spain	English	Substance P NK-1R	Squamous cell carcinomas (114) and adjacent non-tumour epithelium (97)	Larynx (211)	NR	NR	NR	NR	NR	Sx	Mean:40 Range:12-96	O,R,L	University grant	None	IHQ	Clone NR, 1:500	Substance P: Mixed nuclear cytoplasmic<br><br>NK-1R: Mixed membrane cytoplasmic	1	Substance P: LSCC 97.37 ANTE 95.87<br><br>NK-1R: ANTE 92.78
Gonzalez-Moles et al. (b)	2009	Spain	English	Substance P NK-1R	Oral lichen planus (50)	Oral cavity (50)	18/32	Mean: 55.1 Range: 31–79	+10 -40	+12 -38	2001-2004	Sx	0	O,R	University grant	None	IHQ	Clone NR, 1:500	Substance P: Mixed nuclear cytoplasmic<br><br>NK-1R: Mixed membrane cytoplasmic	1	Substance P: 98.0<br><br>NK-1R: 14.0
Brener et al.	2009	Spain	English	Substance P NK-1R	Squamous cell carcinomas (87)	Oral cavity (Tongue 48, Floor of mouth 13, Retromolar trigone 4, Oral mucosa 4, Gingiva 3, Lower lip 2 Vestibular floor 1, mixed 15)	56/17	Mean: 59.8 range: 27-91	NR	NR	NR	Sx	NR	O,R	University grant	None	IHQ	Clone NR, 1:500	Substance P: Mixed nuclear cytoplasmic<br><br>NK-1R: Mixed membrane cytoplasmic	1	Substance P: 77.01<br><br>NK-1R: 50.0 (missing data: 1 cases)
Gonzalez-Moles et al. (c)	2009	Spain	English	Substance P NK-1R	adjacent non-tumour epithelium to squamous cell carcinoma (83)	Oral cavity (83)	51/16	Mean: 60.0 range: 27-91	+38 -29	NR	NR	Sx	NR	O,R	University grant	None	IHQ	Clone NR, 1:500	Substance P: Mixed nuclear cytoplasmic<br><br>NK-1R: Mixed membrane cytoplasmic	1	Substance P: 66.27<br><br>NK-1R: 21.68
Mehboob et al.	2015	Pakistan	English	Substance P	Squamous cell carcinomas (40)	Head and neck (40)	29/11	Mean:53.5 Range:23-80	NR	NR	NR	NR	NR	O,R	University grant	None	IHQ	Clone NR, 1:100	Mixed nuclear cytoplasmic	10	62.5
Isorna et al	2020	Spain	English	Substance P NK-1R	Papillary carcinoma of thyroid (9), Follicular carcinoma of thyroid (8), Medullary carcinoma of thyroid (3)	Thyroid gland (20)	8/12	Mean:46.7 Range:11-76	NR	NR	NR	NR	NR	O,R	University grant	None	IHQ	anti-Substance P antibody: S-1542 1:2000<br><br>anti-NK-1R antibody: SAB-4502913 1:1000	Mixed nuclear cytoplasmic	1	100

LSCC, laryngeal squamous cell carcinoma; OSCC, oral squamous cell carcinoma;ANTE, adjacent non-tumour epithelium to carcinoma; IHC, immunohistochemistry; Sx, surgery; Rt, radiotherapy; CT, chemotherapy; O, observational; R, retrospective; L, longitudinal; NR, not reported.

3. Subgroup meta-analyses on differential expression of substance P/NK-1R in head & neck tumorigenesis and associated factors

Figure S1. Forest plot graphically representing the stratified meta-analysis on differential expression of substance P/NK-1R in head & neck tumorigenesis by malignant behavior (Benign vs. Non-Benign).

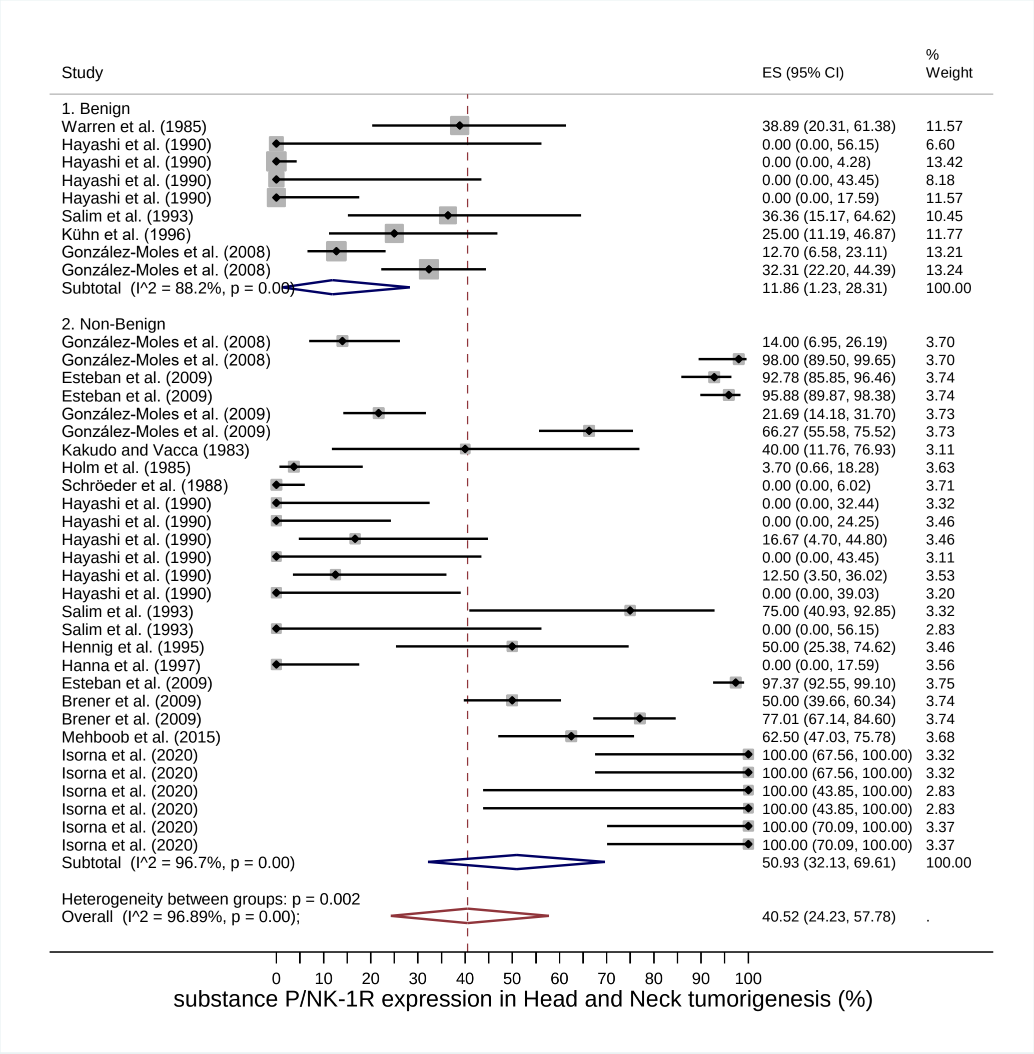

ES, effect size (i.e., pooled proportions expressed as percentage); CI, confidence intervals. Random-effects model, inverse-variance weighting (based on the DerSimonian and Laird method).

Figure S2. Forest plot graphically representing the stratified meta-analysis on differential expression of substance P/NK-1R in head & neck tumorigenesis by malignant behavior (Benign vs. Pre-malignant).

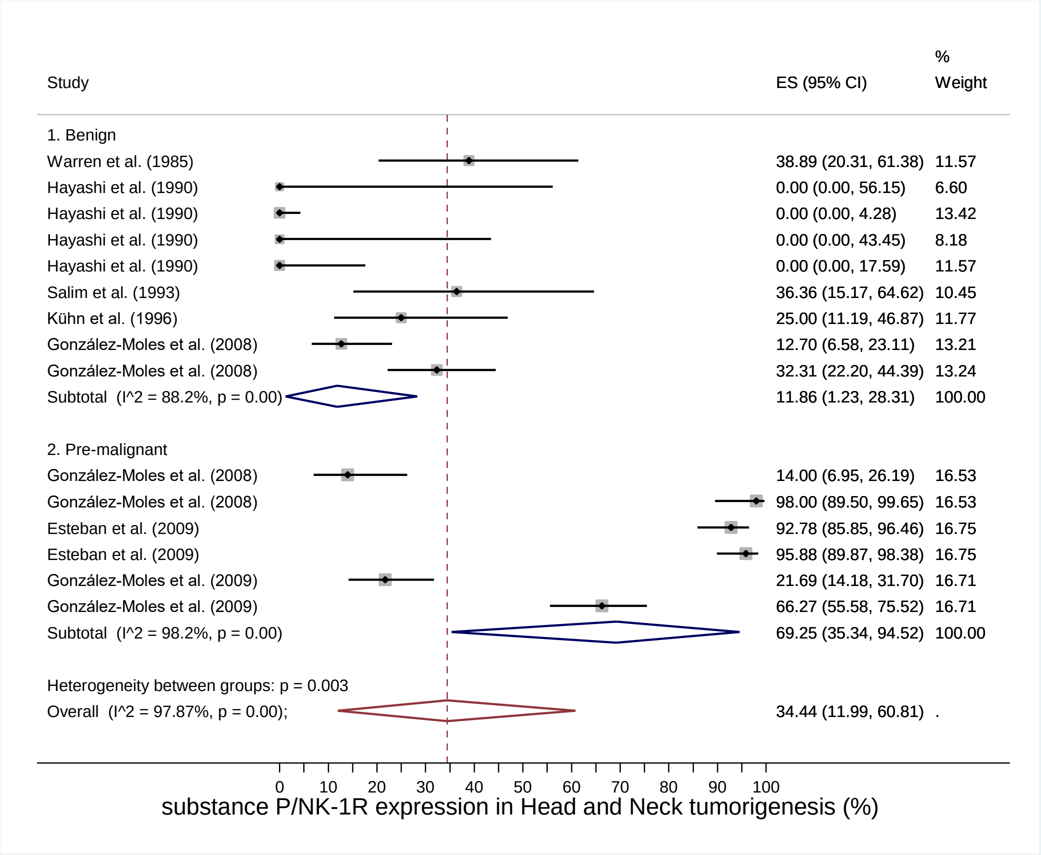

ES, effect size (i.e., pooled proportions expressed as percentage); CI, confidence intervals. Random-effects model, inverse-variance weighting (based on the DerSimonian and Laird method).

Figure S3. Forest plot graphically representing the stratified meta-analysis on differential expression of substance P/NK-1R in head & neck tumorigenesis by malignant behavior (Benign vs. Malignant).

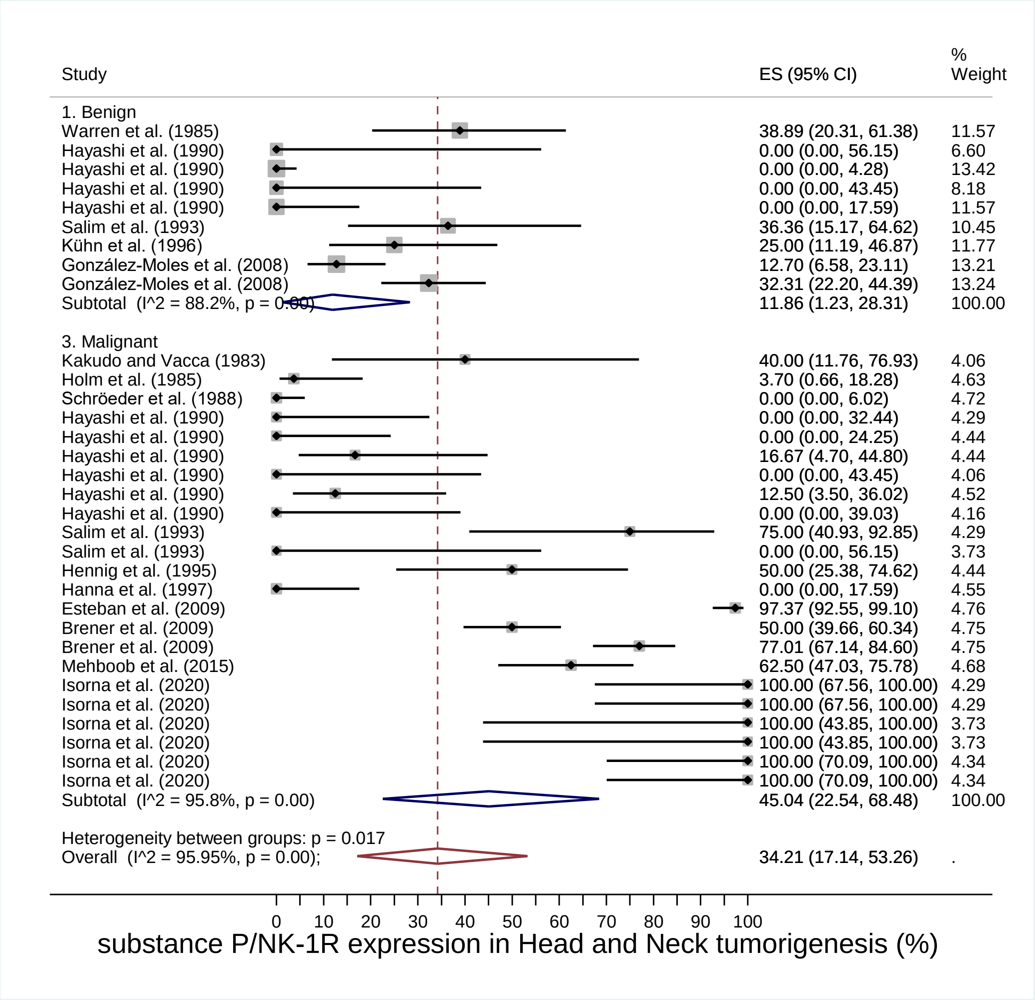

ES, effect size (i.e., pooled proportions expressed as percentage); CI, confidence intervals. Random-effects model, inverse-variance weighting (based on the DerSimonian and Laird method).

Figure S4. Forest plot graphically representing the stratified meta-analysis on differential expression of substance P/NK-1R in head & neck tumorigenesis by malignant behavior (Pre-malignant vs. Malignant).

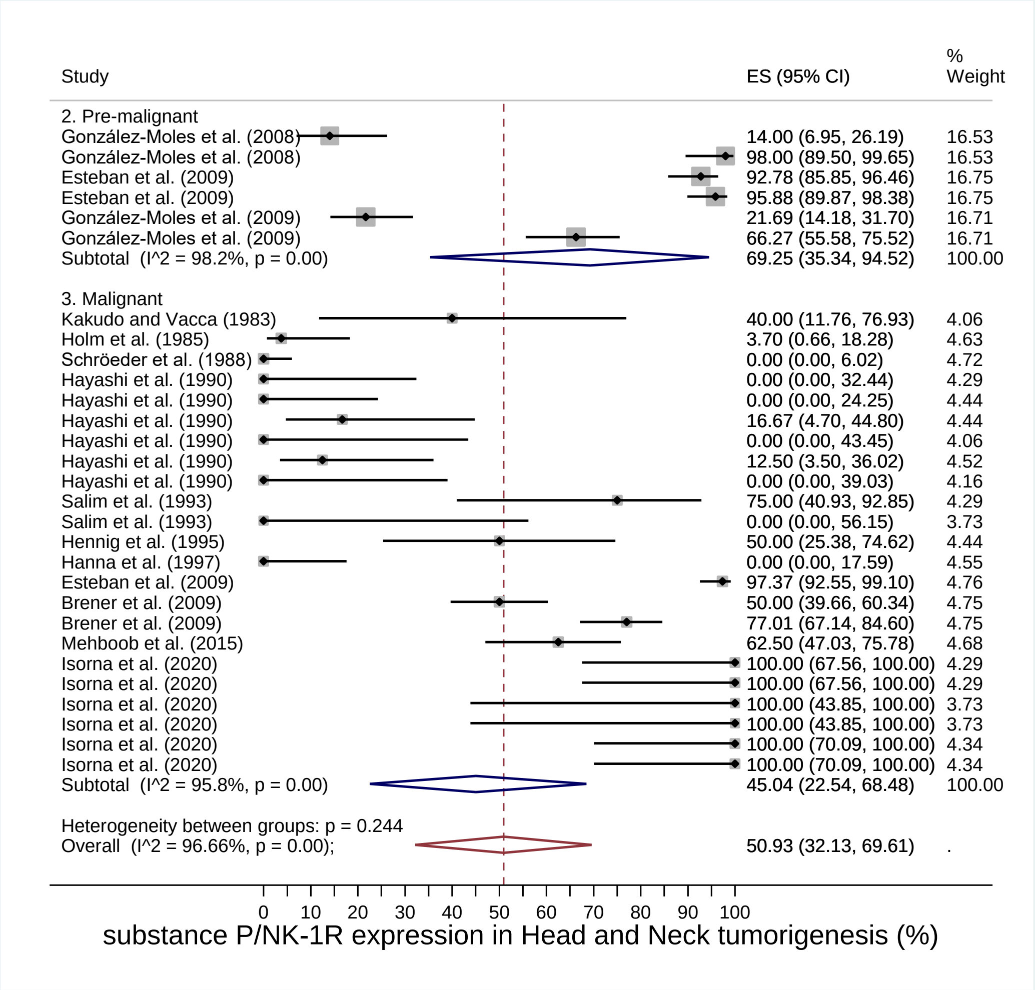

ES, effect size (i.e., pooled proportions expressed as percentage); CI, confidence intervals. Random-effects model, inverse-variance weighting (based on the DerSimonian and Laird method).

Figure S5. Forest plot graphically representing the stratified meta-analysis on differential expression of substance P/NK-1R in benign tumours by geographical area.

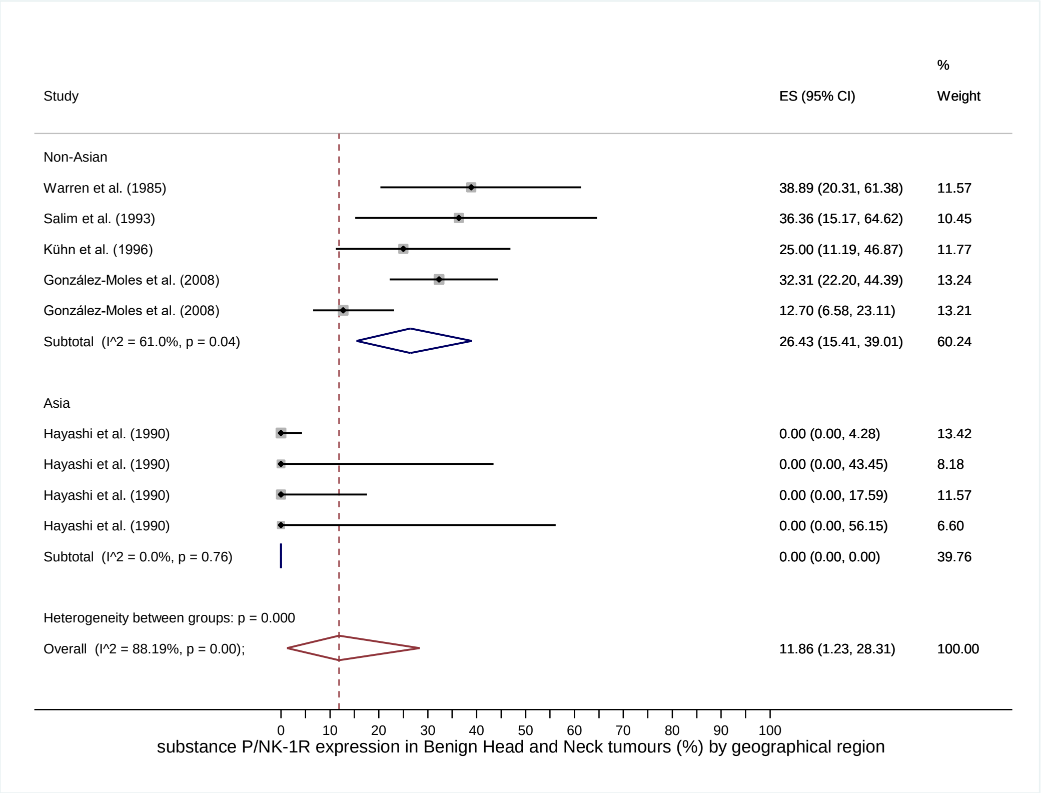

ES, effect size (i.e., pooled proportions expressed as percentage); CI, confidence intervals. Random-effects model, inverse-variance weighting (based on the DerSimonian and Laird method).

Figure S6. Forest plot graphically representing the stratified meta-analysis on differential expression of substance P/NK-1R in benign tumours by anatomical site.

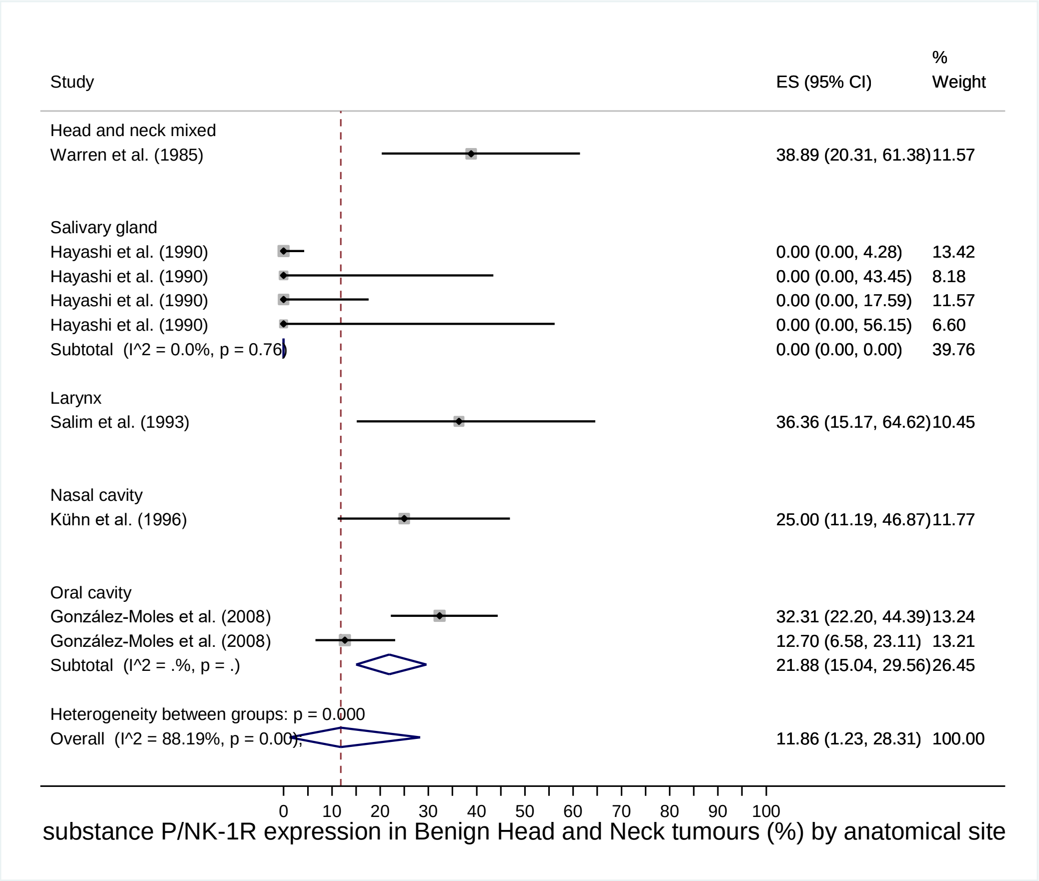

ES, effect size (i.e., pooled proportions expressed as percentage); CI, confidence intervals. Random-effects model, inverse-variance weighting (based on the DerSimonian and Laird method).

Figure S7. Forest plot graphically representing the stratified meta-analysis on differential expression of substance P/NK-1R in benign tumours by histological type.

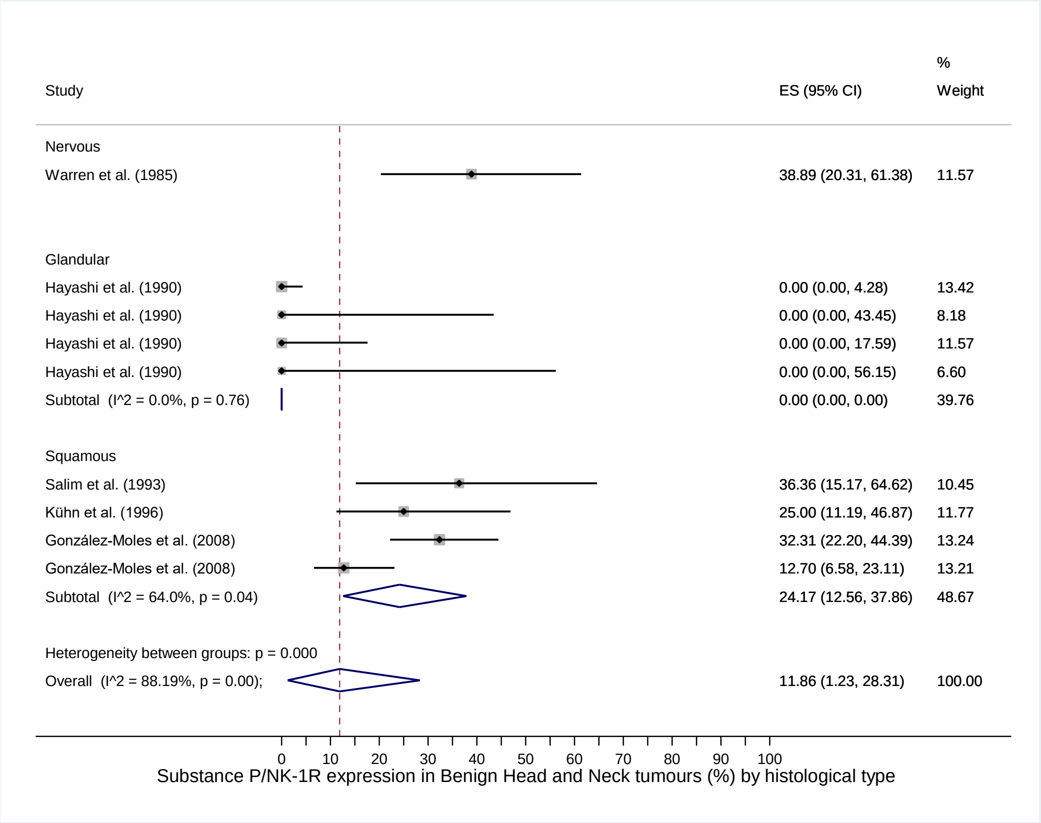

ES, effect size (i.e., pooled proportions expressed as percentage); CI, confidence intervals. Random-effects model, inverse-variance weighting (based on the DerSimonian and Laird method).

Figure S8. Forest plot graphically representing the stratified meta-analysis on differential expression of substance P/NK-1R in benign tumours by biomarker.

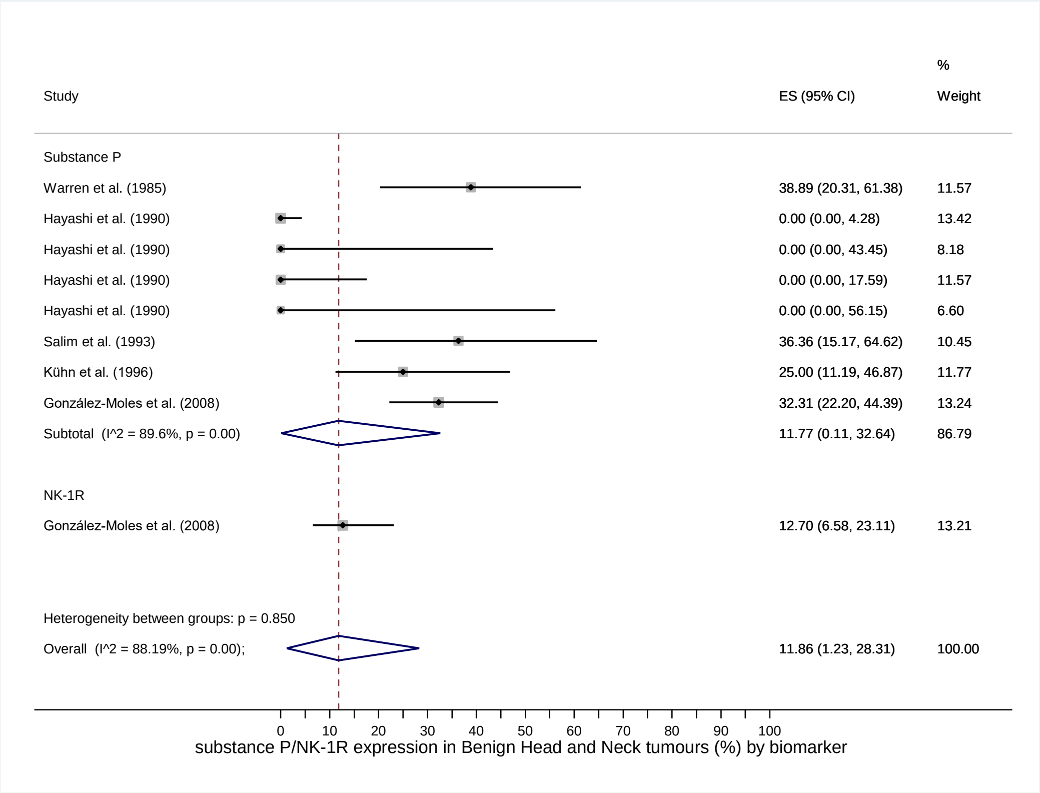

ES, effect size (i.e., pooled proportions expressed as percentage); CI, confidence intervals. Random-effects model, inverse-variance weighting (based on the DerSimonian and Laird method).

Figure S9. Forest plot graphically representing the stratified meta-analysis on differential expression of substance P/NK-1R in pre-malignant tissues by anatomical site.

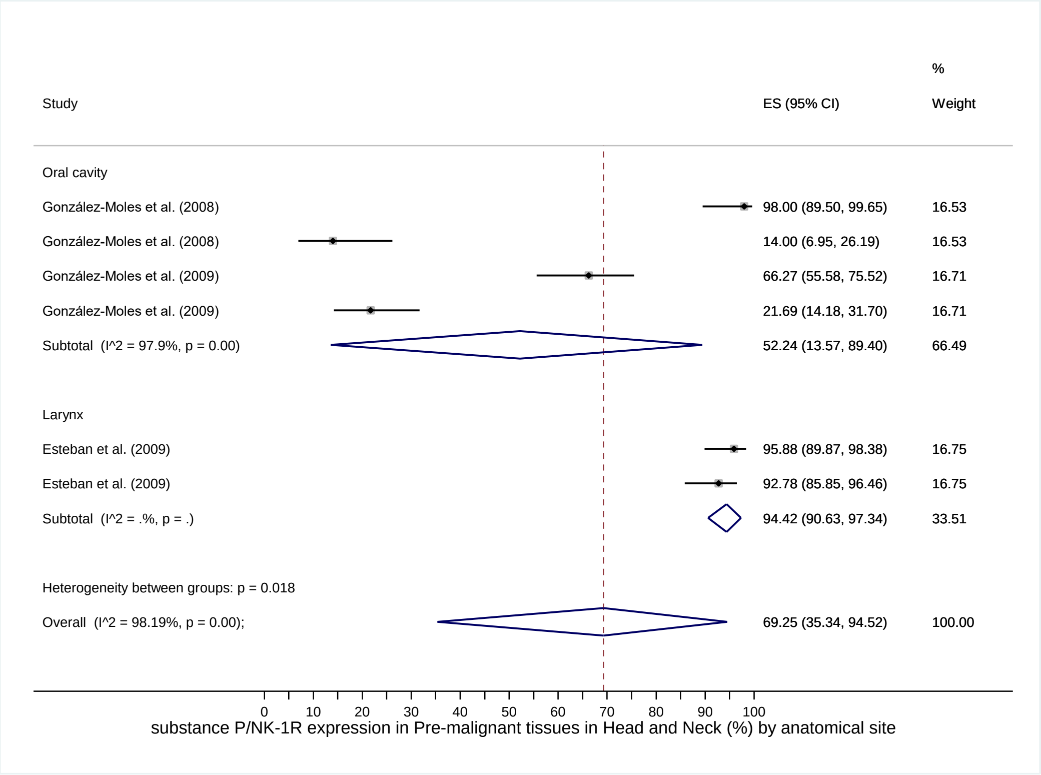

ES, effect size (i.e., pooled proportions expressed as percentage); CI, confidence intervals. Random-effects model, inverse-variance weighting (based on the DerSimonian and Laird method).

Figure S10. Forest plot graphically representing the stratified meta-analysis on differential expression of substance P/NK-1R in pre-malignant tissues by clinical type.

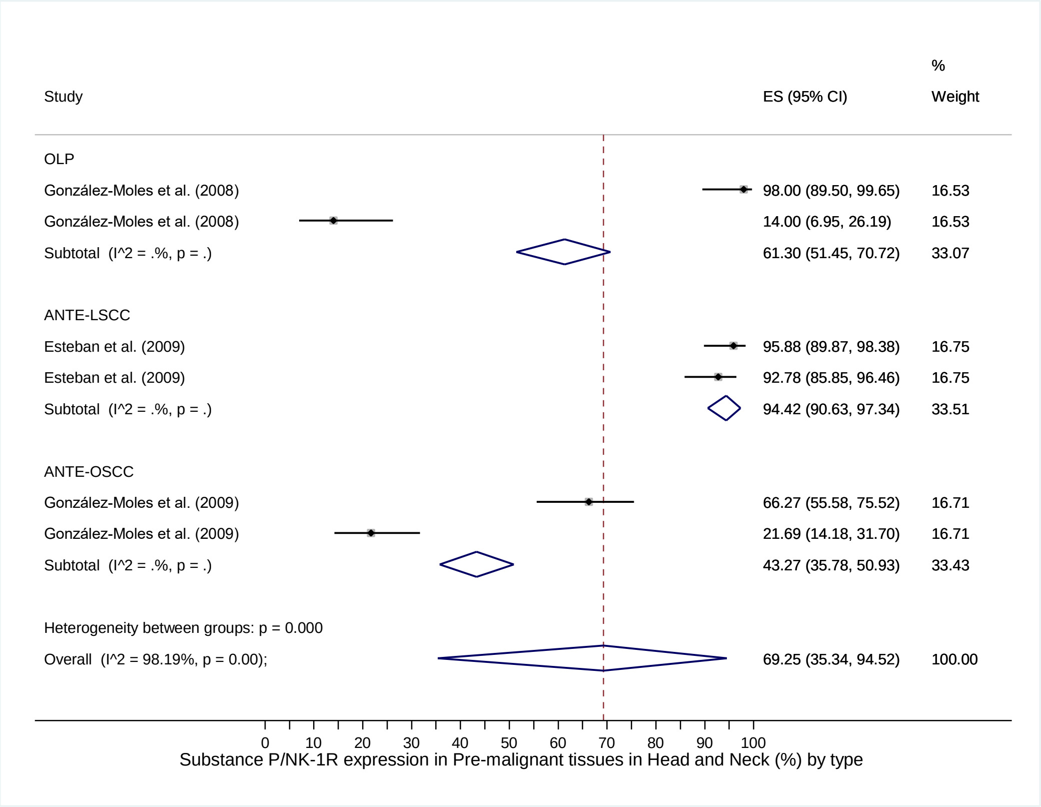

ES, effect size (i.e., pooled proportions expressed as percentage); CI, confidence intervals. Random-effects model, inverse-variance weighting (based on the DerSimonian and Laird method).

Figure S11. Forest plot graphically representing the stratified meta-analysis on differential expression of substance P/NK-1R in pre-malignant tissues by biomarker.

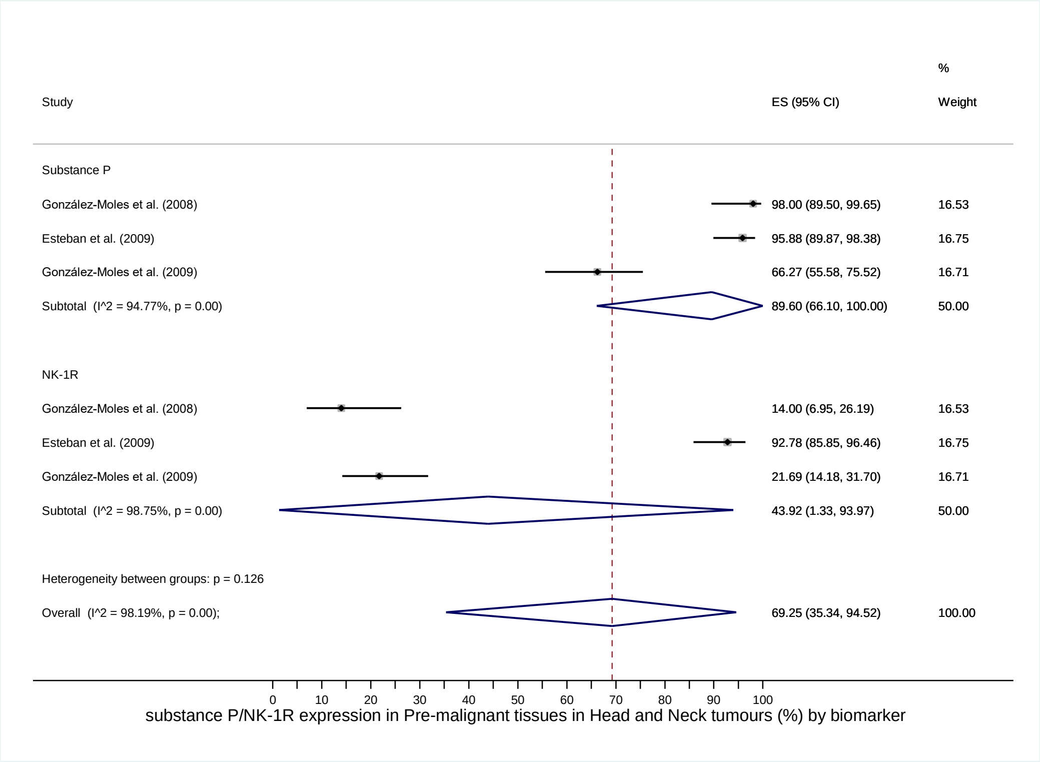

ES, effect size (i.e., pooled proportions expressed as percentage); CI, confidence intervals. Random-effects model, inverse-variance weighting (based on the DerSimonian and Laird method).

Figure S12. Forest plot graphically representing the stratified meta-analysis on differential expression of substance P/NK-1R in malignant tumours by geographical area.

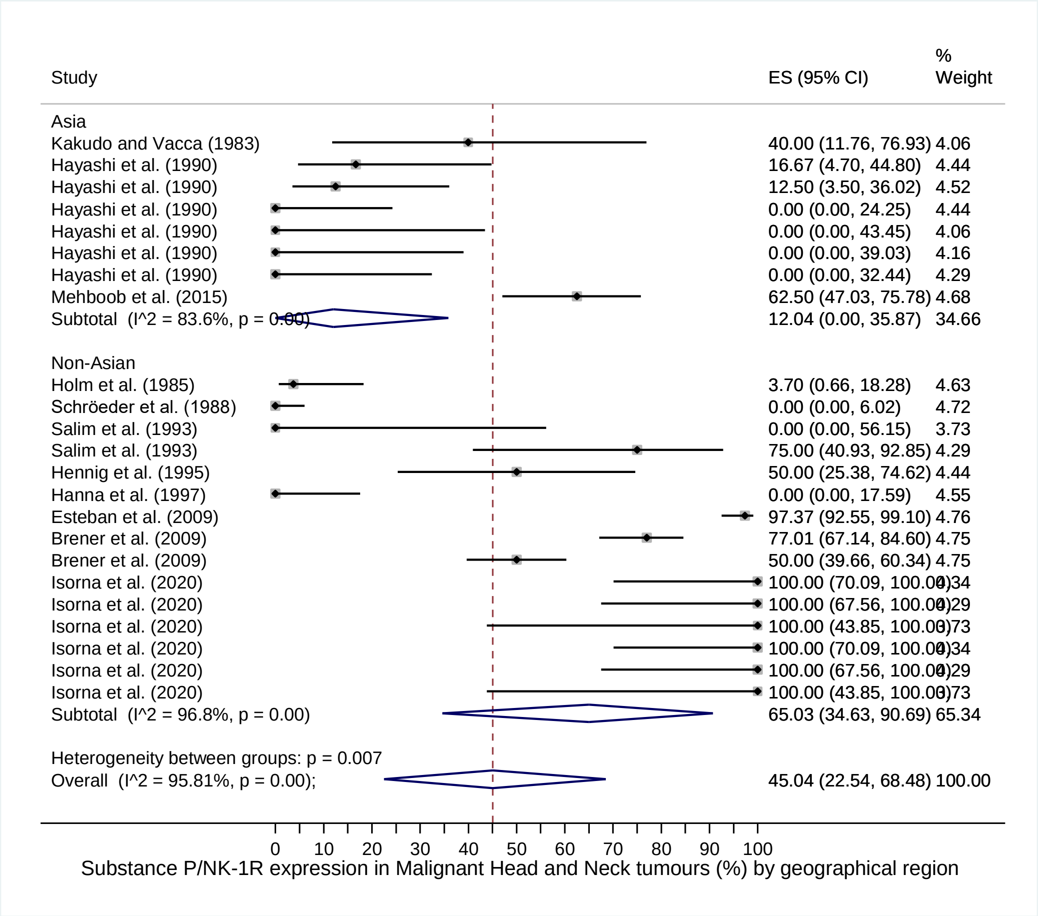

ES, effect size (i.e., pooled proportions expressed as percentage); CI, confidence intervals. Random-effects model, inverse-variance weighting (based on the DerSimonian and Laird method).

Figure S13. Forest plot graphically representing the stratified meta-analysis on differential expression of substance P/NK-1R in malignant tumours by anatomical site.

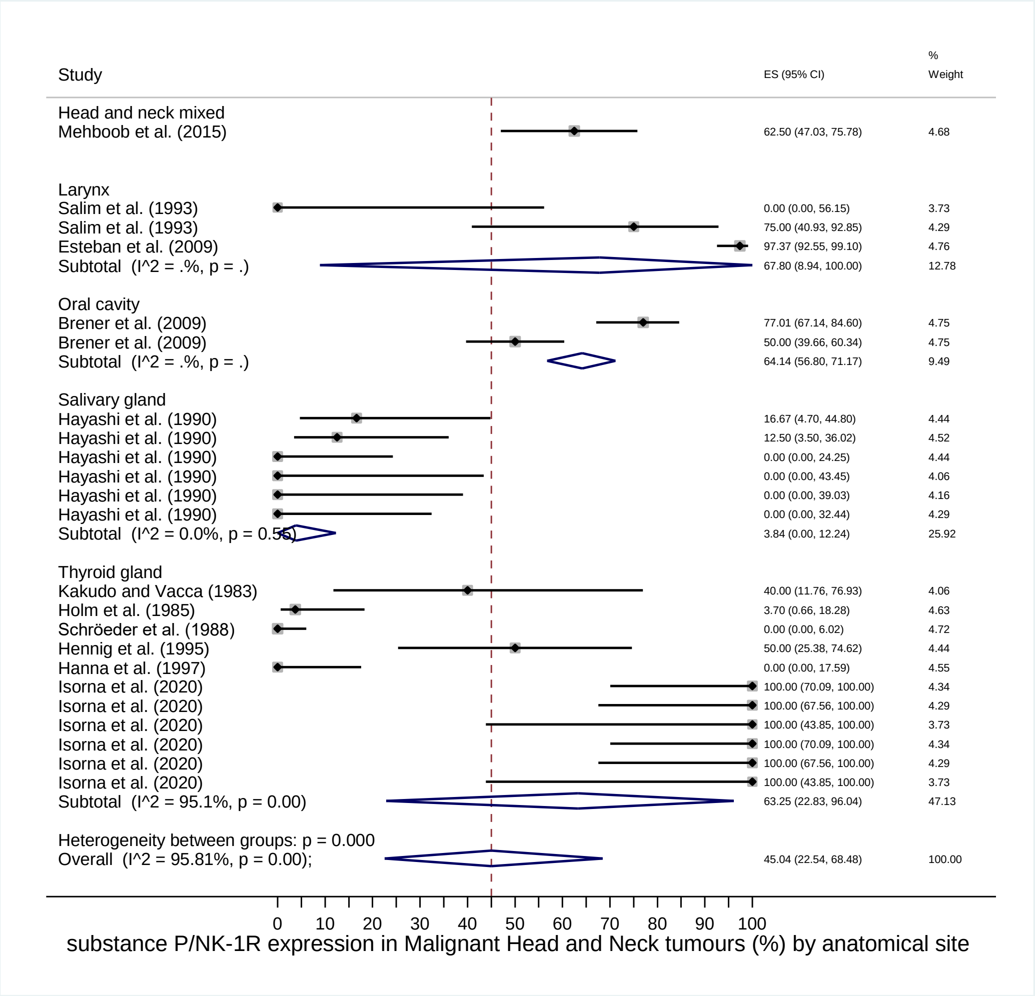

ES, effect size (i.e., pooled proportions expressed as percentage); CI, confidence intervals. Random-effects model, inverse-variance weighting (based on the DerSimonian and Laird method).

Figure S14. Forest plot graphically representing the stratified meta-analysis on differential expression of substance P/NK-1R in malignant tumours by histological type.

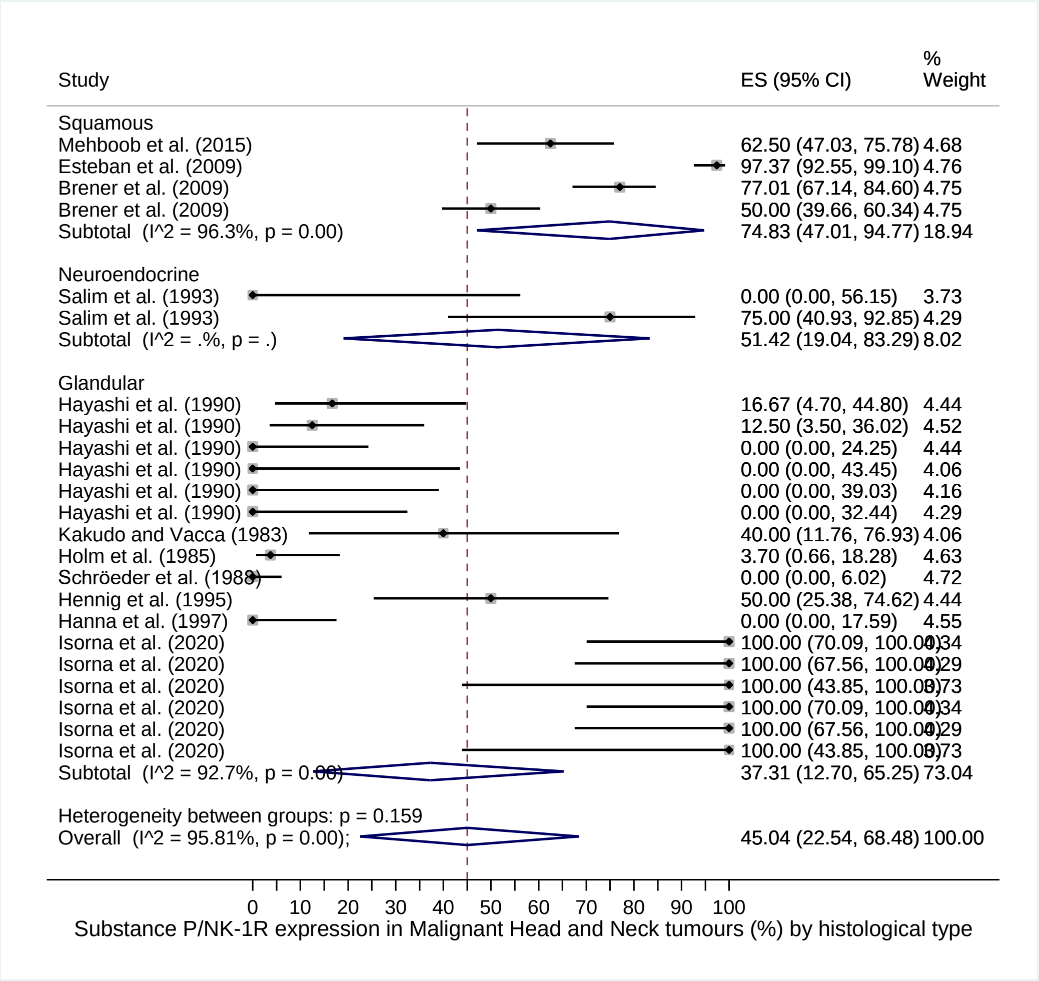

ES, effect size (i.e., pooled proportions expressed as percentage); CI, confidence intervals. Random-effects model, inverse-variance weighting (based on the DerSimonian and Laird method).

Figure S15. Forest plot graphically representing the stratified meta-analysis on differential expression of substance P/NK-1R in malignant tumours by biomarker.

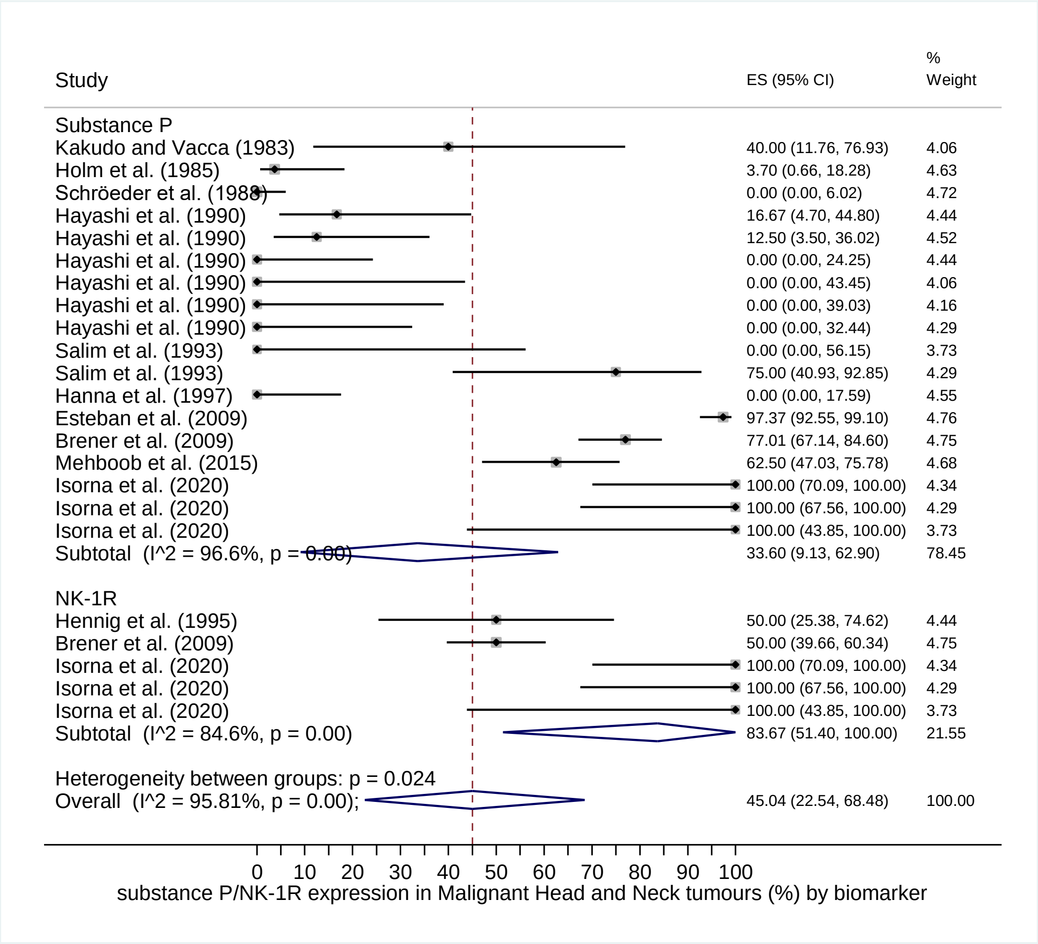

ES, effect size (i.e., pooled proportions expressed as percentage); CI, confidence intervals. Random-effects model, inverse-variance weighting (based on the DerSimonian and Laird method).

4. Sensitivity analysis (leave-one-out method).

4.1 Substance P/NK-1R high expression in head and neck benign tumours.

Table S3. Sensitivity analysis of the studies pooled in the meta-analysis on the expression of substance P/NK-1R in head and neck benign tumours.

Study omitted	Year	Tumour	Biomarker	Estimate	[95% Conf.	Interval]
Warren et al.	1985	Mixed paragangliomas	Substance P	9.03	0.08	25.43
Hayashi et al.	1990	Pleomorphic adenoma	Substance P	16.61	5.97	30.18
Hayashi et al.	1990	Basal cell adenoma	Substance P	13.54	1.77	31.31
Hayashi et al.	1990	Warthin's tumor	Substance P	14.62	1.94	33.44
Hayashi et al.	1990	Oxyphilic adenoma	Substance P	13.49	2.05	30.68
Salim et al.	1993	Laryngeal paraganglioma	Substance P	9.60	0.18	26.30
Kühn et al.	1996	Nasal polyps	Substance P	10.29	0.19	28.09
González-Moles et al.	2008	Keratocystic odontogenic	Substance P	9.18	0.14	25.36
González-Moles et al.	2008	Keratocystic odontogenic	NK-1R	11.77	0.11	32.64
**Combined**				11.86	1.23	28.31

Sensitivity analysis ("leave-one-out" method) of the meta-analysis results, sequentially omitting one study at a time.

4.2 Substance P/NK-1R high expression in head and neck pre-malignant tissues.

Table S4. Sensitivity analysis of the studies pooled in the meta-analysis on the expression of substance P/NK-1R in head and neck pre-malignant tissues.

Study omitted	Year	Tumour	Biomarker	Estimate	[95% Conf.	Interval]
González-Moles et al.	2008	Oral lichen planus	Substance P	61.19	24.26	92.02
González-Moles et al.	2008	Oral lichen planus	NK-1R	79.50	47.60	98.63
Esteban et al.	2009	ANTE-LSCC	Substance P	61.99	24.50	92.79
Esteban et al.	2009	ANTE-LSCC	NK-1R	63.26	24.15	94.33
González-Moles et al.	2009	ANTE-OSCC	Substance P	69.83	28.31	98.01
González-Moles et al.	2009	ANTE-OSCC	NK-1R	78.11	46.65	97.95
**Combined**				69.25	35.34	94.52

Sensitivity analysis ("leave-one-out" method) of the meta-analysis results, sequentially omitting one study at a time.

4.3 Substance P/NK-1R high expression in head and neck malignant tumours.

Table S5. Sensitivity analysis of the studies pooled in the meta-analysis on the expression of substance P/NK-1R in head and neck malignant tumours.

Study omitted	Year	Tumour	Biomarker	Estimate	[95% Conf. Interval]	
Kakudo and Vacca	1983	Medullary carcinoma of thyroid	Substance P	45.25	22.23	69.21
Holm et al.	1985	Medullary carcinoma of thyroid	Substance P	47.78	24.60	71.40
Schröeder et al.	1988	Medullary carcinoma of thyroid	Substance P	48.75	27.48	70.21
Hayashi et al.	1990	Salivary gland adenocarcinoma	Substance P	46.53	23.18	70.57
Hayashi et al.	1990	Salivary gland undifferentiated carcinoma	Substance P	46.88	23.48	70.89
Hayashi et al.	1990	Salivary gland acinic cell carcinoma	Substance P	48.13	24.93	71.70
Hayashi et al.	1990	Salivary gland basal adenoid cystic carcinoma	Substance P	47.54	24.38	71.18
Hayashi et al.	1990	Submandibular salivary gland squamous cell carcinoma	Substance P	47.68	24.49	71.32
Hayashi et al.	1990	Salivary gland mucoepidermoid carcinoma	Substance P	47.88	24.66	71.52
Salim et al.	1993	Laryngeal small cell neuroendocrine carcinoma	Substance P	47.12	24.13	70.68
Salim et al.	1993	Laryngeal large cell neuroendocrine carcinoma	Substance P	43.67	20.75	67.86
Hennig et al.	1995	Medullary carcinoma of thyroid	NK-1R	44.81	21.56	69.11
Hanna et al.	1997	Medullary carcinoma of thyroid	Substance P	48.34	25.25	71.75
Esteban et al.	2009	Laryngeal squamous cell carcinoma	Substance P	41.21	20.75	63.09
Brener et al.	2009	Oral squamous cell carcinoma	Substance P	43.28	19.18	68.88
Brener et al.	2009	Oral squamous cell carcinoma	NK-1R	44.84	20.02	70.86
Mehboob et al.	2015	Head and neck squamous cell carcinoma (mixed)	Substance P	44.14	20.36	69.18
Isorna et al.	2020	Papillary carcinoma of thyroid	Substance P	41.68	19.26	65.74
Isorna et al.	2020	Follicular carcinoma of thyroid	Substance P	41.77	19.33	65.82
Isorna et al.	2020	Medullary carcinoma of thyroid	Substance P	42.66	20.26	66.49
Isorna et al.	2020	Papillary carcinoma of thyroid	NK-1R	41.68	19.26	65.74
Isorna et al.	2020	Follicular carcinoma of thyroid	NK-1R	41.77	19.33	65.82
Isorna et al.	2020	Medullary carcinoma of thyroid	NK-1R	42.66	20.26	66.49
**Combined**				45.04	22.54	68.48

Sensitivity analysis (“leave-one-out” method) of the meta-analysis results, sequentially omitting one study at a time.

5. List of full-text excluded studies with reasons

- In vitro/animal research (n=1)

Muñoz M, Rosso M, Aguilar FJ, González-Moles MA, Redondo M, Esteban F. NK-1 receptor antagonists induce apoptosis and counteract substance P-related mitogenesis in human laryngeal cancer cell line HEp-2. Invest New Drugs. 2008 Apr;26(2):111-8. doi: 10.1007/s10637-007-9087-y. Epub 2007 Sep 29. PMID: 17906845.

- Expression not assessed (n=1)

Misawa K, Kanazawa T, Misawa Y, Imai A, Uehara T, Mochizuki D, Endo S, Takahashi G, Mineta H. Frequent promoter hypermethylation of tachykinin-1 and tachykinin receptor type 1 is a potential biomarker for head and neck cancer. J Cancer Res Clin Oncol. 2013 May;139(5):879-89. doi: 10.1007/s00432-013-1393-5. Epub 2013 Feb 19. PubMed PMID: 23420374.

- Review (n=1)

Esteban F, Muñoz M, González-Moles MA, Rosso M. A role for substance P in cancer promotion and progression: a mechanism to counteract intracellular death signals following oncogene activation or DNA damage. Cancer Metastasis Rev. 2006 Mar;25(1):137-45. doi: 10.1007/s10555-006-8161-9. PMID: 16680578.

- Lack of essential data (n=1)

Otero D, Lourenço SQ, Ruiz-Ávila I, Bravo M, Sousa T, de Faria PA, González-Moles MA. Expression of proliferative markers in ameloblastomas and malignant odontogenic tumors. Oral Dis. 2013 May;19(4):360-5. doi: 10.1111/odi.12010. Epub 2012 Sep 13. PMID: 22970847.

6. List of studies included in this systematic review and meta-analysis.

Isorna I, Esteban F, Solanellas J, Coveñas R, Muñoz M. The substance P and neurokinin-1 receptor system in human thyroid cancer: an immunohistochemical study. Eur J Histochem. 2020 Apr 28;64(2).

Mehboob R, Tanvir I, Warraich RA, Perveen S, Yasmeen S, Ahmad FJ. Role of neurotransmitter Substance P in progression of oral squamous cell carcinoma. Pathol Res Pract. 2015 Mar;211(3):203-7.

Gonzalez-Moles MA, Brener S, Ruiz-Avila I, Gil-Montoya JA, Tostes D, Bravo M, Esteban F. Substance P and NK-1R expression in oral precancerous epithelium. Oncol Rep. 2009 Dec;22(6):1325-31.

Brener S, González-Moles MA, Tostes D, Esteban F, Gil-Montoya JA, Ruiz-Avila I, Bravo M, Muñoz M. A role for the substance P/NK-1 receptor complex in cell proliferation in oral squamous cell carcinoma. Anticancer Res. 2009 Jun;29(6):2323-9.

Esteban F, Gonzalez-Moles MA, Castro D, Martin-Jaen Mdel M, Redondo M, Ruiz-Avila I, Muñoz M. Expression of substance P and neurokinin-1-receptor in laryngeal cancer: linking chronic inflammation to cancer promotion and progression. Histopathology. 2009 Jan;54(2):258-60.

González Moles MA, Esteban F, Ruiz-Avila I, Gil Montoya JA, Brener S, Bascones-Martínez A, Muñoz M. A role for the substance P/NK-1 receptor complex in cell proliferation and apoptosis in oral lichen planus. Oral Dis. 2009 Mar;15(2):162-9.

González Moles MA, Mosqueda-Taylor A, Esteban F, Gil-Montoya JA, Díaz-Franco MA, Delgado M, Muñoz M. Cell proliferation associated with actions of the substance P/NK-1 receptor complex in keratocystic odontogenic tumours. Oral Oncol. 2008 Dec;44(12):1127-33.

Hanna FW, Ardill JE, Johnston CF, Cunningham RT, Curry WJ, Russell CF, Buchanan KD. Regulatory peptides and other neuroendocrine markers in medullary carcinoma of the thyroid. J Endocrinol. 1997 Feb;152(2):275-81.

Kühn AG1, Arnold W. Expression of vasoactive intestinal peptide, substance P and bombesin-flanking peptide in nasal polyps. ORL J Otorhinolaryngol Relat Spec. 1996 Jul-Aug;58(4):229-32.

Hennig IM, Laissue JA, Horisberger U, Reubi JC. Substance-P receptors in human primary neoplasms: tumoral and vascular localization. Int J Cancer. 1995 Jun 9;61(6):786-92.

Salim SA, Milroy C, Rode J, Corrin B, Hamid Q. Immunocytochemical characterization of neuroendocrine tumours of the larynx. Histopathology. 1993 Jul;23(1):69-73.

Hayashi Y, Deguchi H, Nakahata A, Kurashima C, Hirokawa K. Immunopathological study of neuropeptide expression in human salivary gland neoplasms. Pathobiology. 1990;58(4):212-20.

Schröder S, Böcker W, Baisch H, Bürk CG, Arps H, Meiners I, Kastendieck H, Heitz PU, Klöppel G. Prognostic factors in medullary thyroid carcinomas. Survival in relation to age, sex, stage, histology, immunocytochemistry, and DNA content. Cancer. 1988 Feb 15;61(4):806-16.

Holm R, Sobrinho-Simões M, Nesland JM, Gould VE, Johannessen JV. Medullary carcinoma of the thyroid gland: an immunocytochemical study. Ultrastruct Pathol. 1985;8(1):25-41.

Warren WH, Lee I, Gould VE, Memoli VA, Jao W. Paragangliomas of the head and neck: ultrastructural and immunohistochemical analysis. Ultrastruct Pathol. 1985;8(4):333-43.

Kakudo K, Vacca LL. Immunohistochemical study of substance P-like immunoreactivity in human thyroid and medullary carcinoma of the thyroid. J Submicrosc Cytol. 1983 Apr;15(2):563-8.
